# Autoantibodies in hospitalised patients with COVID‐19

**DOI:** 10.1002/cti2.70019

**Published:** 2024-12-26

**Authors:** Eleni Tiniakou, Livia Casciola‐Rosen, Mekha A Thomas, Yuka Manabe, Annukka AR Antar, Mahendra Damarla, Paul M Hassoun, Li Gao, Zitong Wang, Scott Zeger, Antony Rosen

**Affiliations:** ^1^ Division of Rheumatology, Department of Medicine Johns Hopkins University School of Medicine Baltimore MD USA; ^2^ Division of Infectious Diseases, Department of Medicine Johns Hopkins University School of Medicine Baltimore MD USA; ^3^ Division of Pulmonary and Critical Care, Department of Medicine Johns Hopkins University School of Medicine Baltimore MD USA; ^4^ Division of Allergy and Immunology, Department of Medicine Johns Hopkins University, School of Medicine Baltimore MD USA; ^5^ Department of Biostatistics Bloomberg School of Public Health Baltimore MD USA

**Keywords:** anti‐ACE2 IgM, anti‐CD209 IgM, anti‐CD209L IgM, autoantibodies, COVID‐19

## Abstract

**Objectives:**

CD209L and its homologous protein CD209 act as alternative entry receptors for the SARS‐CoV‐2 virus and are highly expressed in the virally targeted tissues. We tested for the presence and clinical features of autoantibodies targeting these receptors and compared these with autoantibodies known to be associated with COVID‐19.

**Methods:**

Using banked samples (*n* = 118) from Johns Hopkins patients hospitalised with COVID‐19, we defined autoantibodies against CD209 and CD209L by enzyme‐linked immunosorbent assay (ELISA). Clinical associations of these antibodies were compared with those of patients with anti‐interferon (IFN) and anti‐angiotensin‐converting enzyme‐2 (ACE2) autoantibodies.

**Results:**

Amongst patients hospitalised with COVID‐19, 19.5% (23/118) had IgM autoantibodies against CD209L and were more likely to have coronary artery disease (44% vs 19%, *P* = 0.03). Antibodies against CD209 were present in 5.9% (7/118); interestingly, all 7 were male (*P* = 0.02). In our study, the presence of either antibody was positively associated with disease severity [OR 95% confidence interval (95% CI): 1.80 (0.69–5.03)], but the association did not reach statistical significance. In contrast, 10/118 (8.5%) had IgG autoantibodies against IFNα, and 21 (17.8%) had IgM antibodies against ACE2. These patients had significantly worse prognosis (intubation or death) and prolonged hospital stays. However, when adjusting for patient characteristics on admission, only the presence of anti‐ACE2 IgM remained significant [pooled common OR (95% CI), 4.14 (1.37, 12.54)].

**Conclusion:**

We describe IgM autoantibodies against CD209 and CD209L amongst patients hospitalised with COVID‐19. These were not associated with disease severity. Conversely, patients with either anti‐ACE2 IgM or anti‐IFNα IgG antibodies had worse outcomes. Due to the small size of the study cohort, conclusions drawn should be considered cautiously.

## Graphical abstract

Amongst patients hospitalised with COVID‐19 infection, we describe IgM autoantibodies against CD209 and CD209L receptors, which are alternative entry receptors for the SARS‐CoV‐2 virus. When comparing these autoantibodies with others known to be associated with COVID‐19 infection, they were not associated with disease severity. Conversely, patients with either anti‐ACE2 IgM or anti‐IFNα IgG antibodies had worse outcomes.

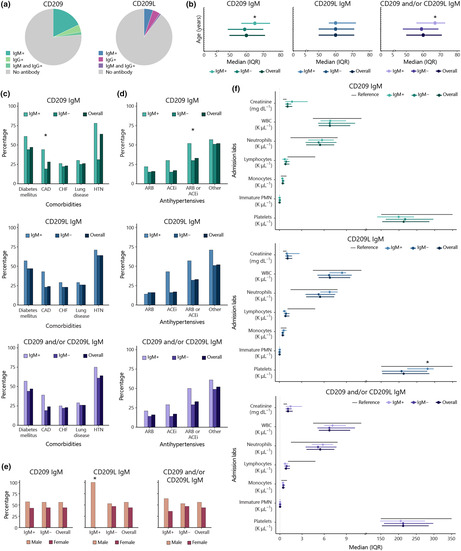

## Introduction

SARS‐CoV‐2 emerged in late 2019 in Wuhan, China,^1^ and has had a momentous global impact resulting in a pandemic. As of September 2023, there have been more than 770 778 396 confirmed cases of COVID‐19, including 6 958 499 deaths, as reported by the World Health Organization (WHO).^2^ Of these, 103 million documented infections and more than 1 million deaths have occurred in the US population.^3^ SARS‐CoV‐2 has evolved significantly since its initial infection of humans, shaped by its new host, natural and induced immunity. The initial focus of SARS‐CoV‐2 on the lung has decreased over time, evolving towards a greater focus on replication in the upper airways.^4^ Worldwide case fatality rates for SARS‐CoV‐2 infection were 7.7% in the first peak of the pandemic (early 2020) and have decreased to < 1% in the summer of 2023, reflecting at least in part significant herd immunity and viral adaptation in the human host.^5^ Understanding the mechanisms and possible biomarkers underlying the severity of the initial presentation of COVID‐19 remains a high priority, as any insights may be of relevance for future Coronavirus pandemics, especially as new variants continue to emerge,^6^, ^7^ and other sporadic illnesses with features similar to severe COVID‐19.

The severity of illness in COVID‐19 was not monomorphic. In some patients, severe disease manifested as an interstitial pneumonia and pulmonary vascular permeability, accompanied by hyperinflammatory features^8^, ^9^ in a subset of these patients. In others, severe disease appeared to be associated with decreased viral clearance.^10^, ^11^, ^12^ In yet others, severe disease demonstrated features of a disseminated coagulopathy.^13^, ^14^ Several studies have implicated a humoral autoimmune response, either pre‐existing or triggered by COVID‐19 infection, as a potential contributing pathway leading to severe outcomes.^15^, ^16^, ^17^ For example, pre‐formed immunoglobulin G (IgG) autoantibodies against type I interferons (IFN), previously demonstrated to be associated with more severe viral infections,^18^, ^19^, ^20^, ^21^ were reported in patients with life‐threatening COVID‐19 infection. These autoantibodies neutralise type I interferon activity and were demonstrated to augment SARS‐CoV‐2 replication in a model system, strongly supporting their role in driving severe outcomes.^22^

An increasing number of reports highlight the significance of autoantibodies linked to endothelial damage as contributors to the severity of COVID‐19 infection. Multiple studies have reported the high prevalence of antiphospholipid autoantibodies.^23^, ^24^, ^25^, ^26^ While the precise role of antiphospholipid antibodies in COVID‐19 outcome is unclear, a distinct group of IgG autoantibodies that target endothelial autoantigens has been identified that could be driving endothelial inflammation and subsequent thrombosis.^27^ IgG and immunoglobulin M (IgM) autoantibodies against neutrophil extracellular traps (NETS) have also been reported in patients with severe COVID‐19. Interestingly, since these have been shown to hinder NET clearance in an *in vitro* model, it is possible that they may lead to immunothrombosis *in vivo*.^28^ Additional insightful information has come from the discovery of novel IgM autoantibodies against angiotensin‐converting enzyme‐2 (ACE2), one of the major host receptors for the coronavirus, in patients with severe COVID‐19.^29^ An especially noteworthy feature of these anti‐ACE2 IgM antibodies is their ability to induce complement activation and endothelium inflammation, particularly in interferon‐exposed endothelial cells.^29^

In the current study, we used banked samples from Johns Hopkins patients hospitalised with COVID‐19 to define additional novel autoantibodies that are elaborated during COVID‐19. Although there is an established link between severe COVID‐19 and endothelial damage through binding to ACE2, the virus can utilise alternative entry receptors through interactions with the spike protein, like CD209L (also known as L‐SIGN) and its homologous protein CD209 (also known as DC‐SIGN), that are highly expressed in virally targeted tissues.^30^, ^31^, ^32^, ^33^, ^34^, ^35^, ^36^, ^37^, ^38^ We, therefore, tested for the presence and clinical features of autoantibodies targeting these receptors and compared these with autoantibodies known to be associated with SARS‐CoV‐2 virus infection. We show that amongst patients hospitalised with COVID‐19, 19.5% have IgM autoantibodies against CD209L, and 5.9% have antibodies against CD209, whereas 6% and 5%, respectively, is the frequency of the IgG isotypes. Surprisingly, these were not associated with disease severity, although anti‐CD209 IgM was significantly more prevalent in hospitalised patients than in patients with mild COVID‐19 infection that did not require hospitalisation. In contrast, patients with either anti‐ACE2 IgM or anti‐IFNα IgG antibodies had worse outcomes.

## Results

### Demographics

We assayed sera from 118 patients admitted to Johns Hopkins Hospital with a confirmed diagnosis of COVID‐19 infection between 20 April 2020 and 14 May 2020 (Table 1). The cohort included patients from 18 to 91 years of age, with a median age of 60 years. Fifty‐six per cent (66/118) of the cohort were male, 26% (31/118) were White and 41% (48/118) were Black. Half of the patients remained in the hospital for 17 days (range: 1–77 days), with a mean stay of 23 days. Regarding other comorbidities typically associated with worse prognosis,^8^ three quarters of patients had elevated body mass index (BMI) (mean BMI: 32.1), 47.5% had a history of diabetes, 64% had hypertension, 23.7% had coronary artery disease (CAD), and 22.9% had congestive heart failure (CHF). Only 26.3% had a prior history of lung disease that could affect the outcome of their infection.

**Table 1:** Patients' clinical characteristics on admission depending on worse hospitalisation outcome of COVID‐19 infection

Patient characteristics	Overall (*N* = 118) Median (IQR)	Min O_2_ (*N* = 34) Median (IQR)	HF O_2_ (*N* = 18) Median (IQR)	Ventilation (*N* = 40) Median (IQR)	Dead (*N* = 16) Median (IQR)
Age (years)	60 (50, 71)	57 (42, 70)	52 (44, 67)	61 (54, 71)	67 (57, 72)
Sex: Male, *n* (%)	66 (56%)	19 (56%)	9 (50%)	24 (60%)	14 (54%)
Ethnicity, *n* (%)					
White	31 (26%)	8 (24%)	3 (17%)	13 (33%)	7 (27%)
Black	48 (41%)	13 (38%)	7 (39%)	17 (43%)	11 (42%)
Other	35 (30%)	11 (32%)	8 (44%)	9 (23%)	7 (27%)
Hispanic	27 (23%)	8 (24%)	6 (33%)	6 (15%)	7 (27%)
BMI (*N* = 106)	30.45 (26.23, 35.18)	30.45 (26.08, 32.75)	30.5 (26.15, 35.3)	31.3 (26.2,40.75)	29.15 (25.3, 36.2)
Comorbidities, *n* (%)					
Diabetes mellitus	56 (47%)	9 (26%)	8 (44%)	24 (60%)	15 (58%)
CAD	28 (24%)	9 (26%)	8 (50%)	10 (25%)	9 (35%)
CHF	27 (23%)	5 (15%)	1 (6%)	13 (33%)	8 (31%)
Lung disease	31 (26%)	6 (18%)	5 (28%)	14 (35%)	6 (27%)
HTN	76 (64%)	18 (53%)	9 (50%)	31 (78%)	18 (70%)
ARB, *n* (%)	19 (16%)	4 (12%)	2 (11%)	6 (15%)	7 (27%)
ACE inhibitors, *n* (%)	21 (17%)	4 (12%)	3 (17%)	10 (25%)	4 (15%)
Either ARB or ACEi, *n* (%)	40 (33%)	8 (24%)	5 (28%)	16 (40%)	11 (42%)
Other antihypertensive medications, *n* (%)	61 (52%)	16 (47%)	5 (28%)	26 (65%)	14 (54%)
Ventilation duration (h) (*N* = 65)	449 (252, 919)	n/a	n/a	502 (275, 1053)	406 (189, 754)
LOS (days), (*N* = 115)	17 (8, 32)	8 (4, 12.25)	12 (7.75, 15.5)	38 (26.5,58.5)	20.5 (12, 32.25)
Admission laboratories					
Creatinine	1.1 (0.8, 1.73)	0.95 (0.7, 1.23)	0.8 (0.7, 1.73)	1.25 (1, 2.2)	1.65 (0.96, 2.08)
WBC (*N* = 117)	6.77 (5.27, 9.5)	6.46 (4.77, 9.28)	7.6 (5.38, 10.23)	6.57 (5.47,8.79)	7.6 (6.08, 13.84)
Neutrophils (*N* = 117)	5.46 (3.6, 7.35)	4.85 (3.16, 6.55)	5.97 (3.43, 7.57)	5.41 (3.95,7.59)	6.53 (4.23, 11.84)
Lymphocytes (*N* = 117)	0.83 (0.62, 1.34)	1.2 (0.67, 1.58)	1.35 (0.8, 1.67)	0.68 (0.49,0.9)	0.85 (0.58, 1.13)
Monocytes (*N* = 102)	0.45 (0.31, 0.67)	0.19 (1.37, 0.75)	0.45 (0.35, 0.74)	0.37 (0.25,0.52)	0.46 (0.3, 0.79)
Immature PMN (*N* = 102)	0.03 (0.02, 0.07)	0.03 (0.02, 0.05)	0.04 (0.02, 0.09)	0.03 (0.02,0.04)	0.045 (0.02, 0.158)
Platelets (*N* = 114)	213 (153, 285)	232 (164 334)	226 (150, 292)	180 (153227)	239 (175, 309)
Medications					
Tocilizumab	16 (14%)	0 (0%)	1 (6%)	10 (25%)	5 (19%)
Steroids	37 (31%)	4 (12%)	2 (11%)	19 (48%)	12 (46%)
Hydroxychloroquine	37 (31%)	2 (6%)	4 (22%)	20 (50%)	11 (42%)
ANA status	56 (48%)	16 (47%)	6 (33%)	25 (62%)	9 (35%)

ACEi, angiotensin‐converting enzyme inhibitor; ANA, antinuclear antibodies; ARB, angiotensin II receptor blocker; BMI, body mass index; CAD, coronary artery disease; CHF, chronic heart failure; HTN, hypertension; IQR, interquartile range; LOS, length of stay; PMN, polymorphonuclear leukocytes; WBC, white blood cell count; WHO, World Health Organization.

*P < 0.05.

Sampling time, calculated as the time between the day of admission and the first positive sample or the last negative sample, was 9 days [interquartile range (IQR): 1–3, 2–22 days]. Slightly more than half of the patients (66/118, 56%) had one sample available (median 5.5 days, IQR 1–15 days), which was drawn within 24 h of onset of the maximum WHO class. The remainder had two to four samples evaluated (median 14 days, IQR 7–31 days). The sampling time for 29/118 (25%) patients was < 3 days, and of those, four (14%) were positive for any anti‐IgM autoantibody (three anti‐CD209 IgM and two anti‐ACE2 IgM). For 52 patients, we had two to four samples available, which we screened for these antibodies. Amongst these, 21 patients were positive for 29 IgM autoantibodies (CD209, CD209L and ACE2). Only eight autoantibodies (five anti‐ACE2 IgM and three anti‐CD209 IgM) became detectable later during their hospitalisation and were not present at the initial sample.

Sixty‐six of 118 patients (56%) had a life‐threatening COVID‐19 infection (dead: 22%, ventilation: 34%). The remainder were either on nasal canula (34/118, 28.8%) or high flow oxygen (18/118, 15.3%), but never required intubation. Patients with severe COVID‐19 infection (dead or ventilated) were more likely to be older (median: 63 vs 55.8 years old, *P* = 0.0121) and had a more prolonged hospital stay (median 28 vs 8.5 days, *P* < 0.0001) when compared to patients with a more favorable outcome (nasal canula or high flow oxygen). While there was no difference in CAD or lung disease prevalence, the patients with higher disease severity were more likely to have diabetes (59% vs 33%, *P* = 0.0054), hypertension (74% vs 52%, *P* = 0.0196) or CHF (32% vs 12%, *P* = 0.0142).

### Patients with COVID‐19 have IgM autoantibodies against CD209 and CD209L

CD209 and its homologous protein CD209L have been shown to directly interact with the spike protein of SARS‐CoV‐2, allowing entry into the host cells.^30^, ^31^, ^32^, ^33^, ^34^, ^35^, ^36^, ^37^ We therefore hypothesised that patients might develop autoantibodies against these important interacting proteins. To address this, we set up ELISAs to assay for the presence of antibodies against CD209 and CD209L in patient sera. Since IgM is the first isotype generated in immune responses, and based on our experience with ACE2 IgM antibodies in COVID‐19 patient studies, we focused on IgM isotypes.

**Figure 1:**
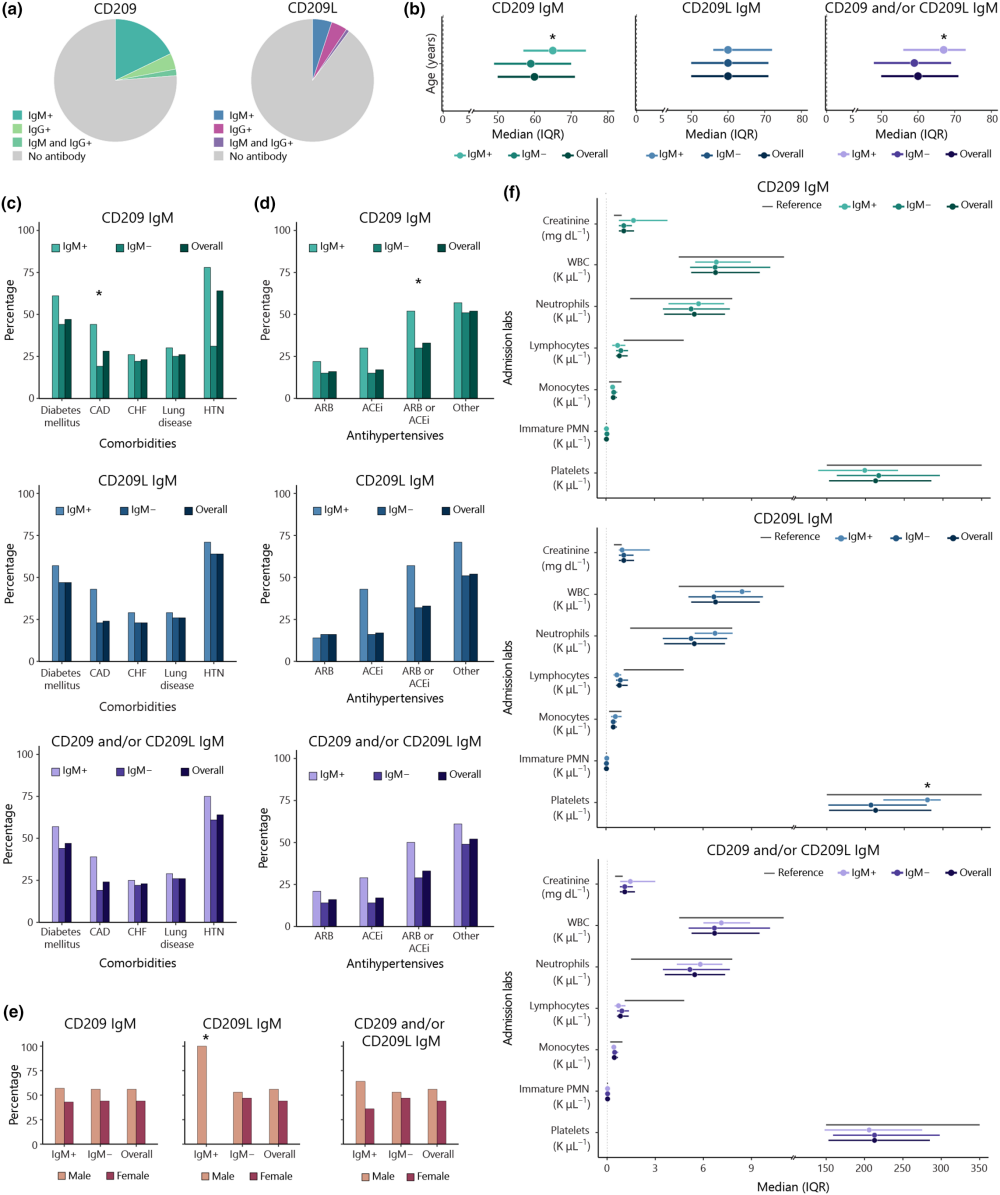
Autoantibodies against CD209 and/or CD209L and their associations with disease characteristics. **(a)** Prevalence of anti‐CD209 IgM and IgG or anti‐CD209L IgM and IgG autoantibodies in disease cohort. **(b)** Median and interquartile range of patient age at admission categorised based on the presence of anti‐CD209 and/or anti‐CD209L IgM antibodies. **(c, d)** Percentage of patients with listed comorbidities **(c)** or given antihypertensive medications **(d)** categorised based on their anti‐CD209 and/or anti‐CD209L IgM autoantibody status. **(e)** Percentage of males and females grouped according to anti‐CD209 and/or anti‐CD209L IgM positivity. **(f)** Median and interquartile range of admission laboratory items of patients with and without anti‐CD209 and/or anti‐CD209L IgM autoantibodies. The normal reference range of item criteria is shown as grey bars. *P ≤ 0.05, **P ≤ 0.01, ***P ≤ 0.001, ****P ≤ 0.0001.

In patients hospitalised for COVID‐19, we found IgM autoantibodies against CD209 in 19.5% (23/118) and CD209L in 5.9% (7/118; Figure 1a). Patients with anti‐CD209 IgM autoantibodies (Supplementary table 1) were more likely to be older (65 vs 59, *P* = 0.05; Figure 1b), to have CAD (44% vs 19%, *P* = 0.03; Figure 1c) and to have been exposed to antihypertensive medications against the renin–angiotensin–aldosterone axis (ACE inhibitors or angiotensin II receptor blockers) (52% vs 30%, *P* = 0.05; Figure 1d), although neither characteristic reached statistical significance. While anti‐CD209L IgM autoantibodies were found less frequently than anti‐CD209 IgM antibodies in the study cohort, it is noteworthy that they were detected exclusively in male patients (Supplementary table 2; 7/7, *P* = 0.02; Figure 1e). Associations with disease severity, pre‐existing conditions or exposures were not identified, except for a trend for higher neutrophils and platelets (6.74 vs 5.26, *P* = 0.09; and 280 vs 207, *P* = 0.05, respectively; Figure 1f).

Despite the high homology of CD209 and CD209L proteins (almost 80%), only two patients were found to possess antibodies that recognised both receptors. Almost a quarter of hospitalised patients had antibodies for either (24%, 28/118) (Supplementary table 3) and were more likely to be older (67 years vs 59 years, *P* = 0.03; Figure 1b). No association with disease severity, pre‐existing conditions or exposures was identified.

We also investigated whether these IgM antibodies transitioned to the IgG subtype and assessed their potential significance. Amongst the patients, 23/118 (19.5%) had IgM antibodies against CD209, but only 7/118 (5.9%) tested positive for anti‐CD209 IgG, with two individuals testing positive for both (Figure 1a). Anti‐CD209L IgG antibodies were found in 6/118 (5.1%) of patients, which was similar to the prevalence of the IgM subtype (7/118, 5.9%), with one individual positive for both subtypes (Figure 1a). No correlation was found between the IgG subtypes and any clinical characteristics, except for the race distribution for anti‐CD209L IgG (Supplementary tables 4 and 5). The presence of either subtype of anti‐CD209 and anti‐CD209L was more common in Caucasians (*P* = 0.02), in older age (68 vs 59 years, *P* = 0.02), and interestingly, it was negatively associated with the presence of anti‐ACE2 IgM antibodies, although the significance was borderline (*P* = 0.05) (Supplementary table 6).

### Anti‐IFNα IgG autoantibodies are associated with severe COVID‐19

**Figure 2:**
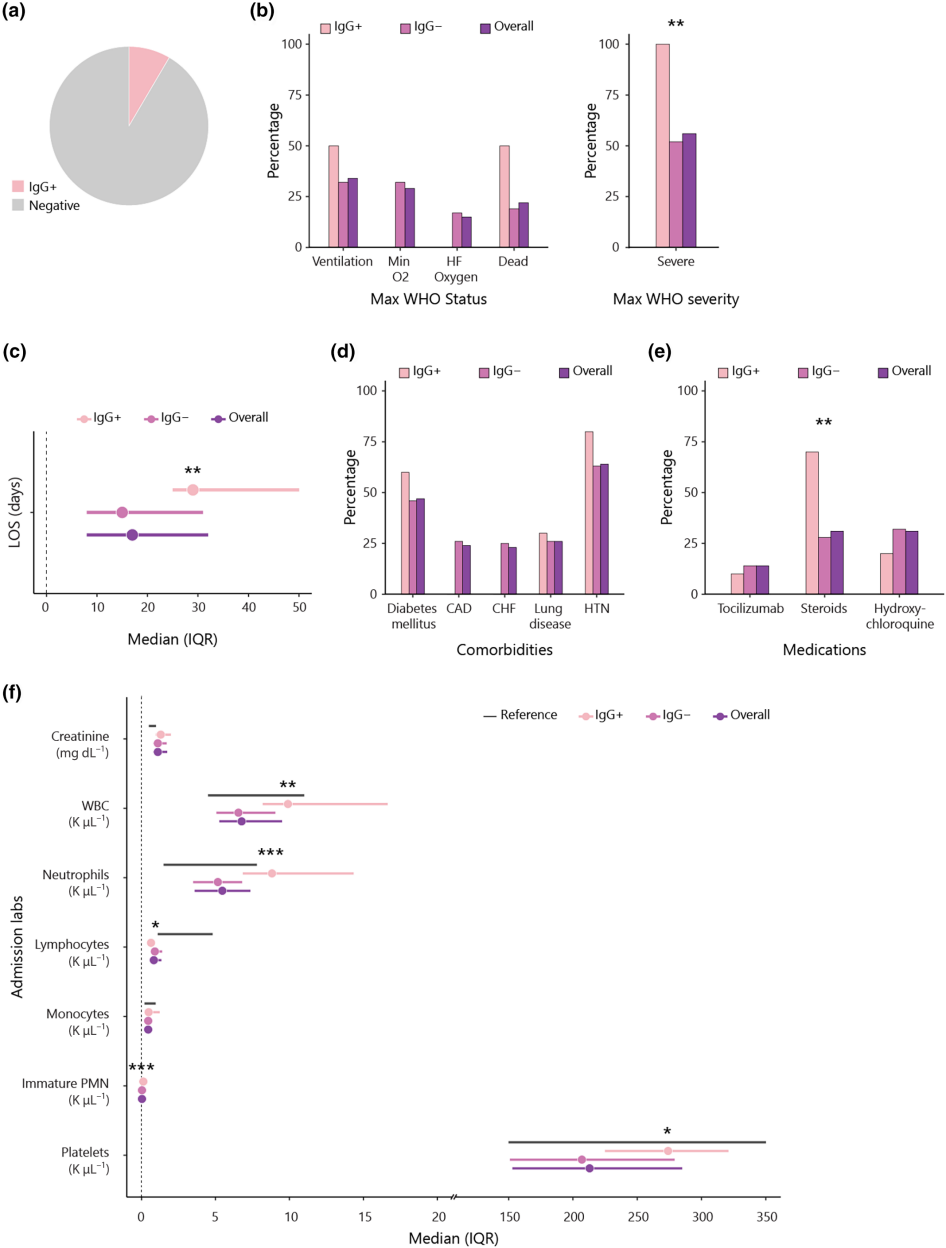
Anti‐IFN‐α IgG antibodies and their associations with disease characteristics. **(a)** Prevalence of anti‐IFN‐α IgG in disease cohort. **(b)** Max WHO status category and Max WHO severity of patients categorised according to the presence of anti‐IFN‐α IgG antibodies. **(c)** Median patient LOS in days grouped according to anti‐IFN‐α IgG positivity. **(d, e)** Percentage of patients with listed comorbidities **(d)** or given anti‐inflammatory medications **(e)** categorised based on their anti‐IFN‐α IgG autoantibody status. **(f)** Median and interquartile range of admission laboratory items of patients with and without anti‐IFN‐α IgG autoantibodies. The normal reference range of item criteria is shown as grey bars. *P ≤ 0.05, **P ≤ 0.01, ***P ≤ 0 0.001, ****P ≤ 0.0001.

We next investigated whether there was any relationship between the autoantibodies above and anti‐IFNα IgG autoantibodies. Ten out of the 118 patients (8.5%, Supplementary table 7; Figure 2a) had autoantibodies against IFNα, similar to previous findings.^22^ It is noteworthy that all 10 of the anti‐IFNα‐positive patients had life‐threatening infection resulting in intubation and/or death (10/66, 15% in the severe COVID‐19 group; Figure 2b). Another striking feature of this group is that their hospital stay was significantly longer (29 vs 15 days, *P* = 0.005; Figure 2c). Surprisingly, they were less likely to have heart disease (CAD, likelihood ratio 5.700, *P* = 0.017; CHF, likelihood ratio 5.466, *P* = 0.019; Figure 2d), reflecting the severity of the viral infection and the treatment guidelines at that time, patients with anti‐IFNα antibodies were more likely to receive steroids (*P* = 0.006; Figure 2e). At the time of admission, the anti‐IFNα‐positive patients had significantly higher levels of multiple inflammatory cell types: white blood cells (WBC) (9.9 vs 6.55, *P* = 0.0013; Figure 2f), neutrophils (8.82 vs 5.16, *P* = 0.0005; Figure 2f), immature polymorphonuclear leukocytes (PMN) (0.12 vs 0.03, *P* = 0.0002; Figure 2f) and platelets (274 vs 207, *P* = 0.02; Figure 2f). While 94% of the patients with autoantibodies against type I IFNs were male in the study by Bastard *et al*.,^22^ in our cohort there was no significant male predominance (6/10, 60%).

The presence of anti‐IFNα IgG autoantibodies is statistically significantly associated with severe COVID‐19 infection [odds ratio OR 95% confidence interval (95% CI): 18.5 (1.00–3.43)]. When adjusting for patients' characteristics on admission using multiple imputation and a Cochran–Mantes–Haenszel test, this association is weakened to 3.19 with 95% CI (0.73–13.9).

### Anti‐ACE2 IgM autoantibodies are associated with severe COVID‐19

**Figure 3:**
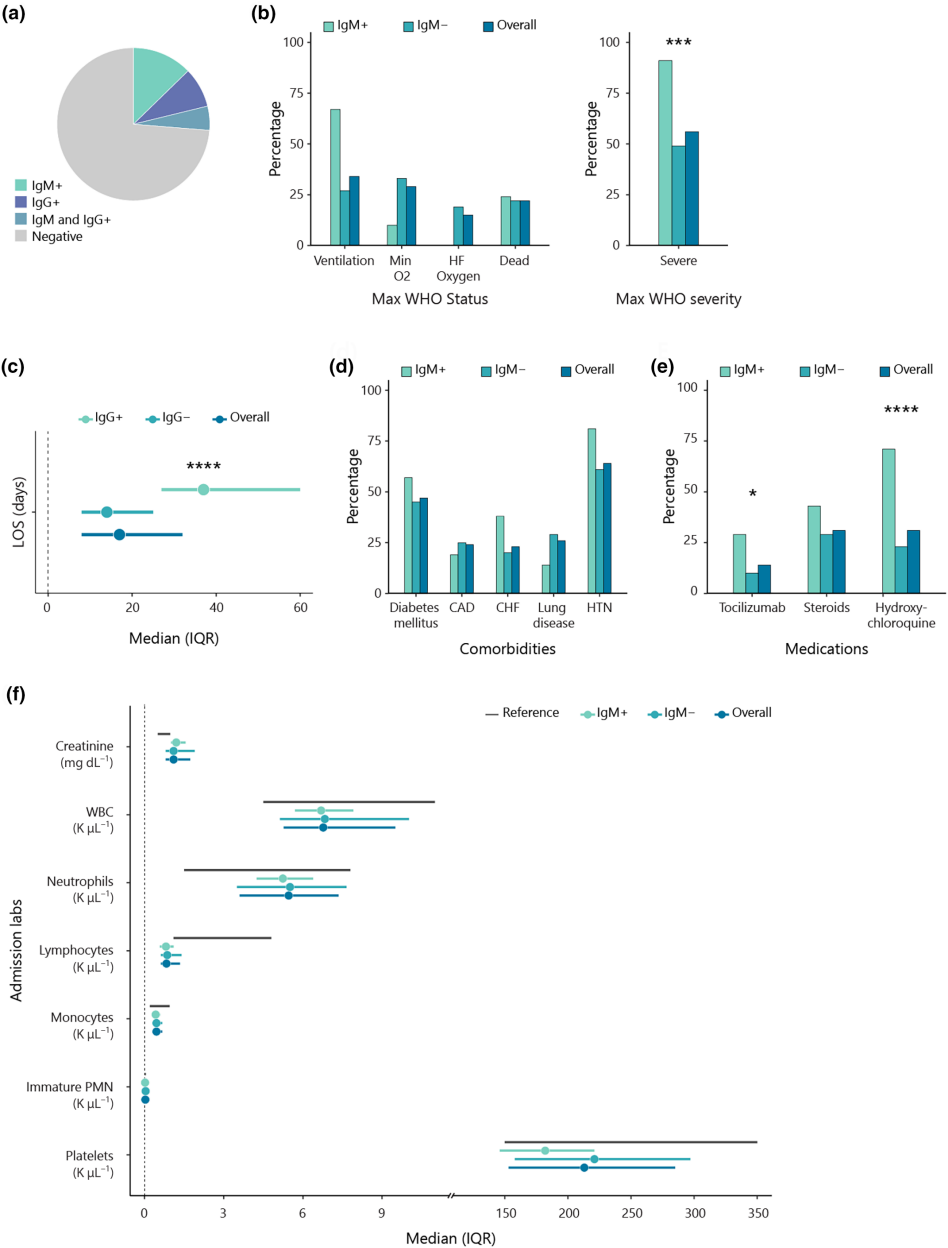
Anti‐ACE2 IgM antibodies and their associations with disease characteristics. **(a)** Prevalence of anti‐ACE2 IgM and IgG in disease cohort. **(b)** Max WHO status category and Max WHO severity of patients categorised according to the presence of anti‐ACE2 IgM antibodies. **(c)** Median patient LOS in days grouped according to anti‐ACE2 IgM positivity. **(d, e)** Percentage of patients with listed comorbidities **(d)** or given anti‐inflammatory medications **(e)** categorised based on their anti‐ACE2 IgM autoantibody status. **(f)** Median and interquartile range of admission laboratory items of patients with and without anti‐ACE2 IgM autoantibodies. The normal reference range of item criteria is shown as grey bars. *P ≤ 0.05, **P ≤ 0.01, ***P ≤ 0 0.001, ****P ≤ 0.0001.

ACE2 acts as the main receptor for SARS‐CoV‐2 allowing the viral entry into host cells, predominantly in lung alveolar and vascular endothelial cells.^38^, ^39^, ^40^ ACE2 IgM antibodies were found in 17.8% (21/118; Supplementary table 8; Figure 3a) of our patient cohort. Similar to our previous report,^29^ patients with anti‐ACE2 IgM antibodies were significantly more likely to develop severe complications of COVID‐19 infection (death or ventilation) compared with the antibody‐negative patients (*P* = 0.0005; Figure 3b), and they were hospitalised for significantly longer time (37 days vs 14 days, *P* < 0.0001) (Figure 3c). There was no statistically significant difference between the two groups as far as comorbidities or admission blood work (Figure 3d and f), but they were more likely to receive tocilizumab (*P* = 0.04), an IL6 inhibitor thought to prevent an inflammatory storm or hydroxychloroquine (*P* < 0.0001, Figure 3e). Interestingly, there was an inverse correlation with anti‐CD209 IgM antibodies (*P* = 0.07). We also assessed the presence of anti‐ACE2 IgG, which amounted to 16% of the cohort (*N* = 104), but there was no difference between severe or moderate disease activity, indicating that it is the IgM isotype that is a significant marker for hospitalised patients (Supplementary table 8).

The presence of anti‐ACE2 IgM autoantibodies is statistically significantly associated with severe COVID‐19 infection [OR (95% CI): 8.69 (1.92–81.2)], and the effect persists even when adjusting for patients' baseline characteristics using the Cochran–Mantes–Haenszel test with multiple imputation [pooled common odds ratio (95% CI): 4.37 (1.40, 13.7)].

### Anti‐ACE2 IgM and anti‐IFN IgG antibodies are associated with disease severity, but not anti‐CD209 IgM or anti‐CD209L IgM antibodies

**Figure 4:**
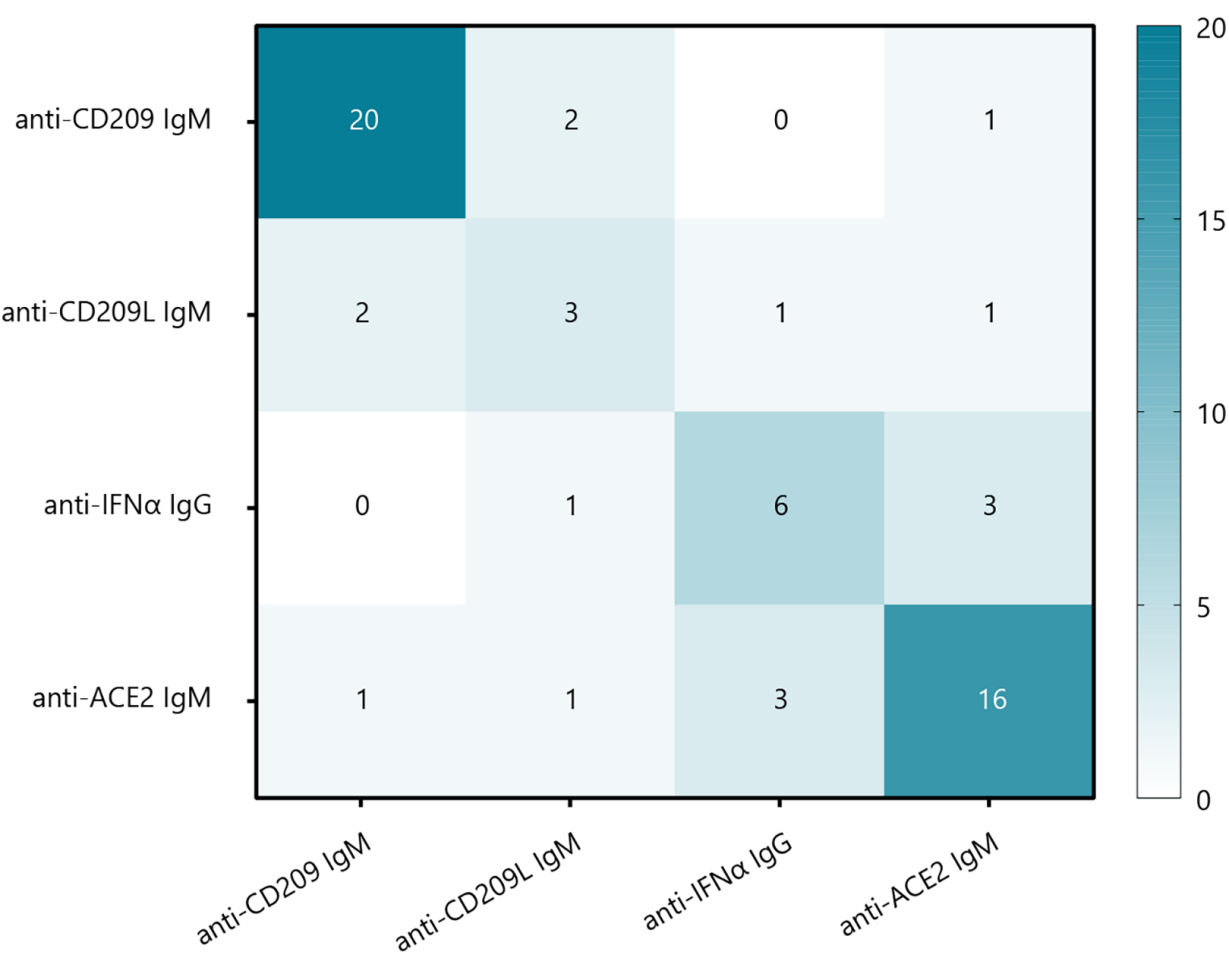
Heatmap depicting autoantibody profiling of cohort.

Amongst the hospitalised patients screened, the lack of overlap in antibody positivity for the different specificities was noteworthy. Only eight patients had more than one autoantibody (Figure 4) (three patients were anti‐ACE2 IgM/anti‐IFNα IgG positive, two patients were anti‐CD209 IgM/anti‐CD209L IgM positive, one patient was anti‐CD209L IgM/anti‐IFNα IgG positive, one was anti‐CD209 IgM/anti‐ACE2 IgM positive, and one patient was anti‐CD209L IgM/anti‐ACE2 IgM positive).

Given the lack of antibody profile overlap, we investigated what parameter(s) would be most associated with disease severity amongst patients hospitalised for COVID‐19 infection, as defined by the worst hospitalisation outcome (namely death or intubation). Using a Cochran–Mantel–Haenszel test with multiple imputation of missing baseline predictors, when patients possess either anti‐CD209 or anti‐CD209L IgM or anti‐IFNα autoantibodies, the presence of anti‐ACE2 IgM was the one significantly associated with COVID‐19 severity [OR (95% CI): 4.14 (1.37, 12.5)]. However, there was no statistically significant association between COVID‐19 severity and anti‐IFNα IgG nor anti‐CD209/CD209L IgM autoantibodies for patients with a similar presentation on admission [OR (95% CI): 3.10 (0.71, 13.47) and 1.39 (0.58, 3.34), respectively].

### Autoantibodies against the receptors for SARS‐CoV‐2 are more prevalent in hospitalised patients infected with COVID‐19 and are associated with disease severity

When investigating the presence of these autoantibodies (anti‐CD209 IgM, anti‐CD209L IgM, anti‐ACE2 IgM and anti‐IFNα IgG) in subjects with COVID‐19 infection with mild disease activity who did not require hospitalisation, we found that anti‐ACE2 IgM (2.9% vs 17.8%, *P* = 0.002), anti‐IFNα IgG (0% vs 8.5%, *P* = 0.013) and anti‐CD209 IgM (5.8% vs 19.8%, *P* = 0.009) were more prevalent in hospitalised patients. Although the prevalence of anti‐CD209L IgM (5.9% vs 1.4%, *P* = 0.144) was similar between the two groups, hospitalised patients had higher titres (*P* = 0.0013) (Supplementary figure 1).

Furthermore, when categorising patients by disease severity – that is, severe (death or intubation), moderate (hospitalised with high flow oxygen or minimal oxygen) and mild (outpatient) – we continued to observe a correlation between disease severity and the presence of anti‐ACE2 IgM, anti‐IFNα IgG and with CD209 IgM (*P* = 0.0307). The prevalence of anti‐CD209 IgG (9.7% vs 5.9%, *P* = 0.55) and anti‐CD209L IgG (4.3% vs 5.1%, *P* > 0.99) did not vary significantly. Moreover, disease severity was positively correlated with the number of autoantibodies (*P* < 0.0001). These findings suggest a strong association between these autoantibodies and the severity of COVID‐19 infection.

When we compared the prevalence of these autoantibodies with a cohort of healthy controls, we observed a similar pattern. The presence of anti‐ACE2 IgM and IgG autoantibodies (3% vs 18%, *P* = 0.04, and 3% vs 14%, *P* = 0.12, respectively), and anti‐CD209 IgM (3% vs 19%, *P* = 0.01) is significantly higher in the hospitalised patients, whereas anti‐CD209 IgG, anti‐CD209L IgG and anti‐CD209L IgM have similar prevalence with the healthy controls. Interestingly, the prevalence of anti‐IFNα IgG (7% vs 8%, *P* = 0.74) antibodies in the healthy controls is not significantly lower, indicating the pre‐existence of these antibodies in the general population.

To evaluate the specificity of these antibodies and decipher their association with COVID‐19 infection or other pulmonary infections requiring hospitalisation, we compared the prevalence of these autoantibodies with a cohort of patients with acute respiratory distress syndrome (ARDS), who represent patients hospitalised due to lung inflammation. While the presence of anti‐CD209L IgM (4% vs 6%, *P* = 0.61) and anti‐CD209 IgM (19.5% vs 13%, *P* = 0.34) is similar between the two cohorts, anti‐ACE2 IgM antibodies (4% vs 18%, *P* = 0.017) are significantly more prevalent in the patients with COVID‐19 infection, indicating a direct association with the SARS‐CoV‐2 virus. Interestingly, the difference in anti‐INFα IgG antibodies (2% vs 8.5%, *P* = 0.12) was not statistically significant between these two hospitalised cohorts, indicating that these antibodies could be responsible for the decreased capacity of these patients to eliminate different infections. Taken together, anti‐ACE2 IgM autoantibodies are unique for COVID‐19 infection and are associated with the most severe form of it.

## Discussion

We have previously shown that a significant percentage (27.2%) of patients with severe COVID‐19 generated IgM antibodies against ACE2, a functional receptor for SARS‐CoV‐2 present on the surface of endothelial and alveolar cells.^29^ We, therefore, investigated whether COVID‐19 also is associated with autoantibodies against alternate receptors for the SARS‐CoV‐2 virus. In this context, we focused on the C‐type lectins, CD209L and CD209, which interact directly with the spike protein of the SARS‐CoV‐2 coronavirus in human cells and have been shown to allow ACE2‐independent viral entry.^30^, ^33^

In this study, we describe two novel IgM autoantibodies against CD209 and CD209L in a cohort of patients hospitalised with COVID‐19. We define the prevalence, clinical profiles and association with disease severity of these new autoantibodies as well as those against ACE2 and IFNα in a cohort of hospitalised patients with COVID‐19. Impressively, 45% (53/118 patients) of the cohort had autoantibodies against either CD209, CD209L, ACE‐2 or IFNα antigens. Of these, 62% (41/53) were found in patients with severe disease compared with 23% (12/53) in patients with mild disease (*P* < 0.0001). These new antibody findings are consistent with previous reports of a highly immunogenic environment that promotes a widespread humoral immune response.^16^, ^41^, ^42^ Additionally, each autoantibody was associated with unique clinical characteristics, with anti‐CD209 IgM being more common in patients with CAD, anti‐CD209L IgM present exclusively in males, and anti‐ACE2 IgM and anti‐IFNα IgG associated with more severe disease. The IgG subtypes of these autoantibodies were less prevalent than their respective IgM subtypes and were not associated with any clinical characteristic. This is similar to our observations with anti‐ACE2 IgG, indicating that the IgM autoantibodies likely have a greater role in COVID‐19 infection. Moreover, when comparing with a cohort of COVID‐19 patients with mild disease (infected during the same time period), the association of anti‐ACE2 IgM, and anti‐IFNα IgG became stronger. In addition, anti‐CD209 IgM was able to distinguish moderate‐worse disease from mild forms of COVID‐19. When comparing with a diverse cohort of patients hospitalised with ARDS due to pneumonia, we did not observe any difference in the prevalence of these autoantibodies, except for anti‐ACE2 IgM. This indicates that anti‐ACE2 IgM is unique for the SARS‐CoV‐2 virus, and that autoantibodies against CD209 and CD209L could be observed in other serious respiratory infections as these proteins act as receptors for other viruses as well (e.g. Ebolavirus, hepatitis C virus, human cytomegalovirus/HHV‐5 and influenza virus).^43^

CD209L is present on alveolar cells, lung endothelial cells and lymph nodes, while CD209 is present on antigen‐presenting cells, such as dendritic cells and macrophages.^30^, ^33^, ^44^, ^45^, ^46^ Despite the greater relevance and prevalence of CD209L to SARS‐CoV‐2 infected tissues, a higher proportion of patients developed antibodies to CD209. The significance of antigen‐presenting cells in COVID‐19 infection is becoming more evident, with recent studies showing that monocytes and macrophages, which can be infected by SARS‐CoV‐2, contribute to the type I IFN response mediated through pyroptosis.^47^, ^48^ Although these studies have emphasised the virus' entry via Fcγ and ACE2 receptors, they have not explored the potential role of CD209. This particular receptor appears to be more focused on transmitting the virus to other vulnerable cells through trans‐infection.^49^ Neither anti‐CD209 nor anti‐CD209L IgM antibodies were associated with disease severity, setting them apart from anti‐ACE2 IgM antibodies. Notably, anti‐ACE2 antibodies play a pivotal role in mediating endothelial damage and dysfunction, contributing to the development of severe COVID‐19 infection. In contrast, neither CD209 or CD209L receptors are found on endothelial cells, potentially accounting for their absence of association with severe disease. Although it did not reach statistical significance (*P* = 0.07), the inverse correlation between anti‐CD209 and anti‐ACE2 autoantibodies is intriguing, and warrants further study.

Also noteworthy is that these antibodies seldom co‐occurred in the same patients (see heat map, Figure 1), despite the extensive homology (> 80%) of the autoantigens,^30^ indicating that these specificities do not read out cross‐reactivity but rather indicate specificity against unique parts of these proteins. Anti‐CD209 IgM antibodies were significantly associated with CAD, and trended towards correlating with advancing age and use of antihypertensive medications targeting the renin–angiotensin–aldosterone pathway. Limited studies have indicated CD209 expression in atherosclerotic plaques and increased expression on dendritic cells of patients with CAD.^50^, ^51^ This has raised the question of whether elevated CD209 expression in the setting of the COVID‐19‐induced inflammatory state triggered the development of these autoantibodies. Moreover, patients with anti‐CD209 IgM antibodies were more likely to have a positive ANA. This observation did not hold true for the other autoantibodies, suggesting the possibility of a dysregulated autoreactive immune response to COVID‐19 in this specific context.^42^

In contrast to anti‐CD209, no clinical associations were noted with antibodies against CD209L. A noteworthy feature of CD209L antibodies was their presence exclusively in male patients in our study cohort (7/7). This discovery parallels the significant overrepresentation of men with autoantibodies against IFN that have been reported by Bastard *et al*,^22^ although this finding was not replicated in our cohort, nor by others.^52^, ^53^, ^54^ Further studies of larger cohorts, including patients with mild infection not requiring hospitalisation, are warranted to investigate the clinical correlations of these antibodies, as well as studies to evaluate the significance of the interaction between SARS‐CoV‐2 and antigen‐presenting cells, like dendritic cells.

Similar to previous studies,^22^ we demonstrated an 8.5% prevalence of anti‐IFNα IgG antibodies in our cohort. Patients with these antibodies all had severe disease, a significantly prolonged hospital stay and laboratory evidence of inflammation, while they tended to have a negative correlation with predisposing factors, like CAD or CHF (*P* = 0.11). This possibly reflects the fact that these are pre‐existing autoantibodies, which become functionally significant during an aggressive viral infection (e.g. COVID‐19), since they can neutralise secreted IFNs, thereby facilitating infection of susceptible cells and promoting viral replication.^22^ However, it is impressive that the correlation with disease severity diminishes when adjusting for patients' characteristics on admission in the presence of anti‐ACE2 IgM autoantibodies.

While anti‐ACE2 IgM antibodies were also associated with severe disease, their genesis is most likely mediated in the setting of COVID‐19 infection given their isotype (IgM) and their specificity against the entry receptor of the virus. They were not associated with any traditional risk factors of severity for COVID‐19 infection (such as age, sex, BMI and diabetes). When correcting for all other antibodies and comorbidities, the presence of these antibodies continued to confer a significantly higher risk for prolonged hospitalisation and worse outcome (intubation or death).

There are several limitations to this study. While this study focused on patients with COVID‐19 requiring hospitalisation, the small size of the cohort is a limitation and any conclusions drawn should be considered with caution. Additional studies are warranted to gain insight into the potential roles of these autoantibodies in segregating patients and their trajectories across the broader spectrum of COVID‐19. Information about the date of exposure/symptom onset was not collected. As the best possible alternative to address the timing issue, we calculated the sampling time from the day of admission to either the first positive sample or the last negative sample in cases with multiple samples. We recognise that this likely underestimates the true sampling time from exposure or symptom onset. Moreover, for a quarter of our cohort, samples were available only within the first 3 days of hospitalisation, and only four of those tested positive for any IgM autoantibodies. This may further contribute to an underestimation of the true prevalence of these IgM isotype autoantibodies.

We described the anti‐ACE2 IgM autoantibodies in the setting of COVID‐19 infection in a previous study,^29^ where we showed that these antibodies were able to activate complement and caused functional changes in endothelial cells in a tissue‐engineered pulmonary microvessel model, thus indicating their potential pathogenetic role in severe COVID‐19 infection. We examined these antibodies in this study as well, as our goal was to describe the full panel of autoantibodies associated with COVID‐19 infection in moderate–severe disease (hospitalised patients), and to find whether multiple antigens were targeted in a single patient. We do not know whether anti‐CD209 and anti‐CD209L antibodies have functional consequences; additional future studies to define this are warranted.

This is the first description of autoantibodies against alternate receptors for the SARS‐CoV‐2 viral entry – CD209 and CD209L – in a cohort of hospitalised patients with COVID‐19 infection of varying severity. Our finding that nearly half (53/118) of these patients had autoantibodies (alone or in various combinations) against CD209, CD209L, ACE‐2 or IFNα is consistent with a highly immunogenic environment fostered by COVID‐19 infection. Both anti‐IFNα IgG and anti‐ACE2 IgM were associated with disease severity, consistent with a potential functional role in enhancing viral replication and inflammation. This could occur either through neutralising the capacity of IFNα to impede SARS‐CoV‐2 infection of target cells, or by inducing complement‐mediated immunothrombosis. Considering the persistent risk of an impending pandemic due to the ever‐changing SARS‐CoV‐2 virus, these autoantibodies may be clinically useful as prognostic indicators of disease severity. In addition, they may provide mechanistic insights into COVID‐19 pathogenesis.

## Methods

### Study cohort: Patients hospitalised with COVID‐19

Full details of the patient cohorts studied have been described previously.^29^ A subset of these samples was used in this study. Briefly, the study cohort consisted of 118 inpatients with (1) a confirmed diagnosis of COVID‐19 and (2) remnant specimens banked in the Johns Hopkins COVID‐19 Remnant Specimen Biorepository. This is an opportunity sample that includes patients with length of stay ≥ 3 days between 20 April 2020 and 14 May 2020, and included a broad range of disease severities with similar numbers of patients with moderate (52 patients) and severe disease (66 patients). Diagnosis of COVID‐19 was defined as detection of SARS‐CoV‐2 using any PCR test with an Emergency Use Authorization from the US Food and Drug Administration. Selection and frequency of other laboratory testing were determined by treating physicians. The primary clinical data source was JH‐CROWN, a Johns Hopkins Medicine COVID‐19 registry that integrates all clinical data for COVID‐19 patients.^8^

### Disease and healthy control cohorts

We also examined a cohort of patients who were infected with SARS‐CoV‐2 during the same time period, but remained outpatient.^55^ The cohort of non‐hospitalised patients with mild COVID‐19 infection consisted of 69 participants with blood samples collected within 25 and 90 days after symptom onset. Three patients presented to the emergency room after enrolment for COVID‐19 hypoxia or other symptoms, but none of these patients was hospitalised. We utilised samples from 50 patients with pneumonia‐associated (primary) ARDS; these were selected from patients enrolled in the ARDSNet Fluids and Catheters Treatment Trial (FACTT).^29^ Sera from *N* = 35 healthy adults were studied as controls.

All clinical information and sera were collected under IRB‐approved protocols, and all patients and healthy controls provided written informed consent to participate.

Patient outcomes were defined by the WHO COVID‐19 disease severity scale. For this study, we combined adjacent WHO classes, dividing the study population into three groups according to maximum WHO severity: (1) patients who did not require hospitalisation, (2) patients who did not require mechanical ventilation (WHO classes 3–5), but severe enough to hospitalised [this moderate disease activity group (*N* = 52) included patients on high flow oxygen (*N* = 18) and with minimal O2 requirements (*N* = 34)] and (3) patients who required mechanical ventilation. This severe group (*N* = 66) included both those who survived [(WHO classes 6 and 7, *N* = 42) or died (WHO class 8, *N* = 24)]. Serum samples were selected for timing within 24 h of the onset of the maximum WHO class; when multiple samples were available, the specimen closest to the WHO class onset was used.

### Autoantibody assays

#### ELISAs to detect IgM antibodies against ACE2, CD209 and CD209L

ELISA plate wells were coated overnight with purified recombinant human protein diluted in PBS (50 ng per well ACE2 (Abcam, Cambridge, UK); 200 ng per well CD209 (ProSci Incorporated, Poway, CA, USA) or 50 ng per well CD209L [Sino Biological, Beijing, China)]. For each serum assayed, duplicate wells were coated with protein, and an adjacent well was incubated overnight with PBS for background determination. Wells were washed with PBS plus 0.1% Tween (PBST), then blocked with 3% milk/ PBST. Primary antibody incubations were performed by diluting sera 1:200 (anti‐ACE2 and anti‐CD209L) or 1:100 (anti‐CD209) in 1% milk/ PBST (overnight, 4°C). Wells were then washed with PBST, followed by incubation with horseradish peroxidase (HRP)‐labelled anti‐human IgM (Heavy chain‐specific; Jackson ImmunoResearch, West Grove, PA, USA # 109‐035‐043) diluted 1:5000 in 1% milk/ PBST (1 h, room temperature (RT)). Colour was developed with SureBlue peroxidase reagent (KPL, Gaithersburg, MD, USA). Reactions were terminated by adding HCl, and absorbances were read at 450 nm. For each ELISA type, the same positive reference serum with an optical density reading in the linear range was included on every plate and all absorbances were calibrated relative to this. Cut‐offs for assigning antibody positivity were determined by assaying sera from healthy controls. The mean + 3 SD of these values [calibrated OD units of 0.340 (anti‐ACE2 IgM), 0.179 (anti‐CD209 IgM) and 0.201 (anti‐CD209L IgM)] was taken as the positive cut‐off for each of these antibodies.

#### Anti‐IFNα, CD209 and CD209L IgG ELISAs

These were performed as described above, with the following modifications. Wells were coated with purified recombinant human CD209 or CD209L (both 50 ng per well, and purchased from the sources detailed above), or IFNα (200 ng per well, Sigma). The concentration of Tween in PBST was 0.05%. Blocking was performed with 5% BSA/PBST (for anti‐CD209 and anti‐CD209L ELISAs) or 3% blotto/PBST (anti‐IFNα ELISA). Sera and secondary antibodies were diluted with 1% BSA/PBST (anti‐CD209 and CD209L ELISAs) or 1% blotto/PBST (anti‐IFNα ELISA). Sera were assayed at 1:100 dilution (anti‐IFNα) or 1:200 dilution (anti‐CD209 and ‐CD209L) for 90–120 min, at room temperature. The secondary antibody was HRP‐labelled anti‐human IgG (Jackson ImmunoResearch, West Grove, PA, USA # 109‐036‐088) and was used diluted 1: 10000. The cut‐off (calibrated OD units) for assigning antibody positivity, determined as described above, was 0.075 (anti‐IFNα ELISA), 0.424 (anti‐CD209 ELISA) and 0.529 (anti‐CD209L ELISA). All ELISAs were set up, optimised and validated by coating wells with recombinant protein and detecting with the following appropriate commercially available antibodies: anti‐IFNα (R&D Systems, mouse monoclonal cat # MAB93452), anti‐CD209L (Invitrogen, rabbit monoclonal # MA5‐47780) and anti‐CD209 (Invitrogen, rabbit monoclonal # MA5‐29099).

### Statistical analysis

Demographic and clinical characteristics were analysed using descriptive statistics. Subgroup comparisons were conducted using the Fisher exact test, or the Wilcoxon rank sum test when appropriate. These statistical computations were carried out using JMP17. The statistical assessments were two‐sided, and statistical significance was established at *P*‐values below 0.05 for all analyses. Patients' baseline characteristics were defined in terms of whether they had BMI over 30 at admission, whether patients were older than 75 years at admission, vital signs and laboratory measurements at admission, including WBC count, respiratory rate, SaO2/FiO2, pulse, temperature, D‐Dimer, C‐reactive protein, absolute lymphocyte count and eGFR.

When quantifying the association between severity and autoantibodies, Fisher's exact test was used in the unadjusted analysis. In order to calculate covariate‐adjusted odds ratio, we used multiple imputation^56^ to account for missing covariate data, propensity scores to simplify the adjustment process and principal component analysis to reduce to two the dimensions of laboratory and vital signs measures. To address missing data in patients' baseline characteristic, in brief, we imputed five complete datasets using multiple imputation with chained equations.^57^ Due to the limited sample size, laboratory and vital sign measures were subsequently summarised in terms of the first two components of a principal component analysis. For each imputed dataset, we assigned estimated each person's propensity score: the expected probabilities of harbouring a positive autoantibody of interest as a function of covariates using logistic regression, for each patient as their propensity scores through logistic regressions. These propensity scores were then used to create five equally sized propensity strata based on quantiles of the expected probabilities for each autoantibody. A covariate‐adjusted odds ratio was calculated using Cochran–Mantes–Haenszel statistics with Haldane–Anscombe correction for small sample sizes for each of the five randomly imputed dataset. Rubin's rule was applied to generate the final odds ratio and 95% CI. In reporting the results, this approach was used to estimate the severity‐autoantibody odds ratios with different levels of adjustments for other factors: (1) no adjustment (no imputation needed); (2) adjustment for baseline patient characteristics; and (3) adjustment for baseline patient characteristics and the presence of the other autoantibodies.

Given the sample size, adjustment for covariates and distribution of autoantibodies, the power to detect an association of severity and CD209/CD209L ranges from 41 to 95% as the true odds ratio varies from 3.0 to 10 (Supplementary figure 2).

## Conflict of interest

The authors declare no conflicts of interest.

## Author contributions

**Eleni Tiniakou:** Data curation; formal analysis; investigation; methodology; writing – original draft; writing – review and editing. **Livia Casciola‐Rosen:** Conceptualization; data curation; formal analysis; funding acquisition; investigation; methodology; project administration; supervision; validation; writing – review and editing. **Mekha A Thomas:** Formal analysis; visualization; writing – review and editing. **Yuka Manabe:** Formal analysis; writing – review and editing. **Annukka AR Antar:** Formal analysis; writing – review and editing. **Mahendra Damarla:** Formal analysis; writing – review and editing. **Paul M Hassoun:** Formal analysis; funding acquisition; writing – review and editing. **Li Gao:** Formal analysis; writing – review and editing. **Zitong Wang:** Formal analysis; methodology; writing – review and editing. **Scott Zeger:** Formal analysis; methodology; writing – review and editing. **Antony Rosen:** Conceptualization; data curation; formal analysis; funding acquisition; investigation; methodology; project administration; resources; supervision; validation; writing – review and editing.

## Supporting information


Supplementary Information

## Data Availability

The data and specimens utilised were part of JH‐CROWN: The COVID‐19 PMAP Registry, the Johns Hopkins COVID‐19 Remnant Specimen and Johns Hopkins CCPSEI Repositories. These resources are based on the contribution of many patients, research teams and clinicians, and were funded by the University and Hopkins inHealth, the Johns Hopkins Precision Medicine Program. The data that support the findings of this study are available on request from the corresponding author. The data are not publicly available due to privacy or ethical restrictions.
