# Supplementary material for: Autoantibodies in hospitalised patients with COVID‐19
Source: Clin Transl Immunology. 2024 Dec 26;13(12):e70019. doi: 10.1002/cti2.70019 (PMC11671454; doi:10.1002/cti2.70019)
Supplement: Supplementary file 1 — Supplementary Information [file CTI2-13-e70019-s001.docx]

**SUPPORTING INFORMATION**


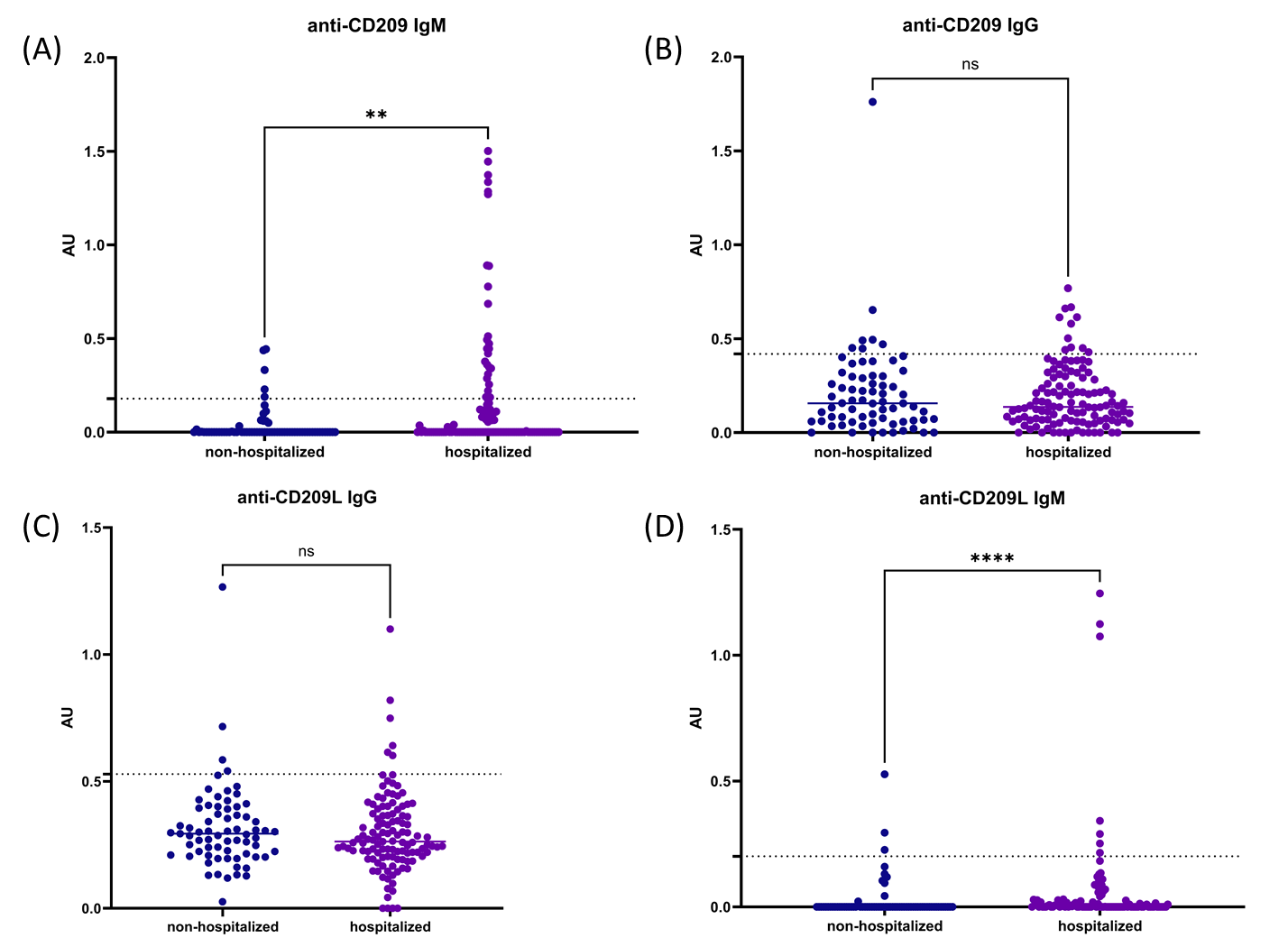


(a)

(b)

(c)

(d)

**Supplementary figure 1.** Antibody titers (AU, arbitrary units) for **(a)** anti-CD209 IgM, **(b)** anti-CD209 IgG, **(c)** anti-CD209L IgM and **(d)** anti-CD209L IgG between non-hospitalized and hospitalized patients with COVID-19 infection.


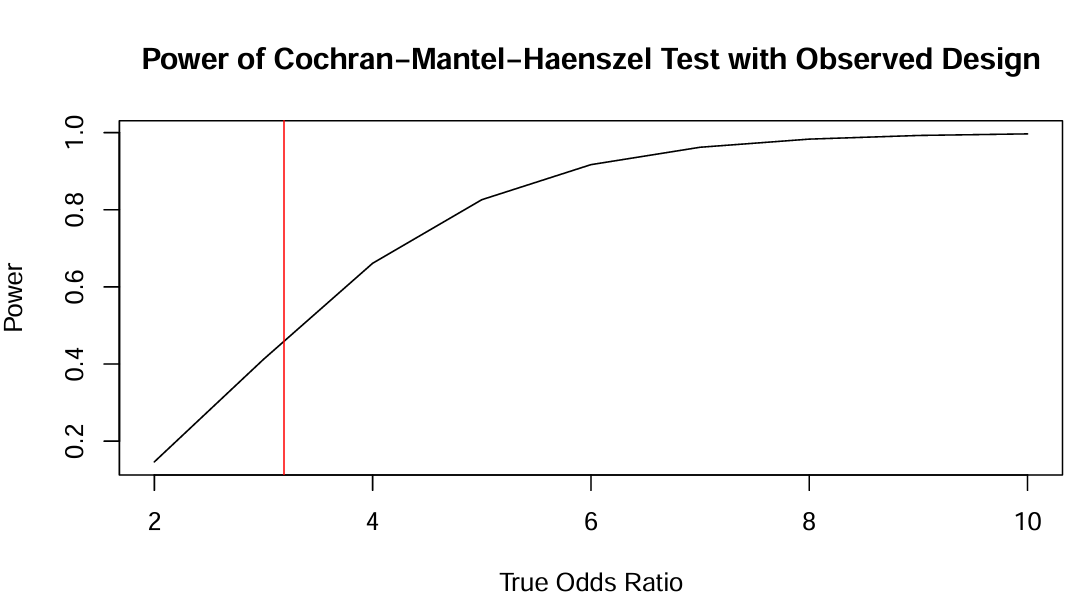


**Supplementary figure 2.** Power curve against true odds ratio using multiple imputation and a Cochran-Mantel Haenzel test when adjusting for patients’ characteristics on admission.

**Supplementary table 1.** Comparison of patients’ clinical characteristics based on the presence of anti-CD209 IgM autoantibody.

| **Patient Characteristics** | **Overall (n=118)**  **Median (IQR)** | **Detectable Anti-CD209 IgM (n=23)** | **Non-detectable IgM (n=95)** | ***P*-value** |  |
| --- | --- | --- | --- | --- | --- |
| **Age (years)** | 60 (50, 71) | 65 (57,74) | 59 (49,70) | 0.0503 |  |
| **Sex: Male, n (%)** | 66 (56%) | 13 (57%) | 53 (56%) | 1.0 |  |
| **Ethnicity, n (%)** |  |  |  | 0.22 |  |
| White | 31 (26%) | 10 (44%) | 21 (22%) |  |  |
| Black | 48 (41%) | 6 (26%) | 42 (44%) |  |  |
| Other | 35 (30%) | 0 (0%) | 3 (3%) |  |  |
| Hispanic | 27 (23%) | 7 (30%) | 28 (30%) | 0.72 |  |
| **BMI (n=106)** | 30.45 (26.23, 35.18) | 31.05(25.45,34.48) | 30.4(26.23,36.02) | 0.94 |  |
| **Max WHO Status** |  |  |  | 0.37 |  |
| Ventilation | 40 (34%) | 10 (43%) | 30 (32%) |  |  |
| Min O2 | 34 (29%) | 4 (17%) | 30 (32%) |  |  |
| HF Oxygen | 18 (15%) | 5 (22%) | 13 (14%) |  |  |
| Dead | 26 (22%) | 4 (17%) | 22 (23%) |  |  |
| **Max WHO severity, severe** | 66 (56%) | 14 (61%) | 52 (55%) | 0.65 |  |
| **Ventilation duration (hours) (n=65)** | 449 (252, 919) | 361(153,1010) | 466(274,890) | 0.56 |  |
| **LOS (days), (n=115)** | 17 (8, 32) | 18(13,39) | 17(8,33) | 0.48 |  |
| **Comorbidities, n (%)** |  |  |  |  |  |
| Diabetes mellitus | 56 (47%) | 14 (61%) | 42 (44%) | 0.17 |  |
| CAD | 28 (24%) | 10 (44%) | 18(19%) | 0.03^*^ |  |
| CHF | 27 (23%) | 6 (26%) | 21 (22%) | 0.78 |  |
| Lung disease | 31 (26%) | 7 (30%) | 24 (25%) | 0.61 |  |
| HTN | 76 (64%) | 18 (78%) | 58 (31%) | 0.15 |  |
| **ARB, n (%)** | 19 (16%) | 5 (22%) | 14 (15%) | 0.53 |  |
| **ACE inhibitors, n (%)** | 21 (17%) | 7 (30%) | 14 (15%) | 0.12 |  |
| **Either ARB or ACEi, n (%)** | 40 (33%) | 12 (52%) | 28 (30%) | 0.0504 |  |
| **Other antihypertensive medications, n (%)** | 61 (52%) | 13 (57%) | 48 (51%) | 0.65 |  |
| **Admission Labs** |  |  |  |  |  |
| Creatinine | 1.1 (0.8, 1.73) | 1.7(0.8,3.8) | 1.1(0.8,1.6) | 0.11 |  |
| WBC (n=117) | 6.77 (5.27, 9.5) | 6.82(5.52,8.94) | 6.75(5.2,10.15) | 0.99 |  |
| Neutrophils (n=117) | 5.46 (3.6, 7.35) | 5.72(3.87,7.3) | 5.26(3.51,7.65) | 0.79 |  |
| Lymphocytes (n=117) | 0.83 (0.62, 1.34) | 0.72(0.39,1.19) | 0.92(0.63,1.36) | 0.15 |  |
| Monocytes (n=102) | 0.45 (0.31, 0.67) | 0.41(0.29,0.5) | 0.48(0.32,0.69) | 0.17 |  |
| Immature PMN (n=102) | 0.03 (0.02, 0.07) | 0.03(0.02,0.05) | 0.06(0.02, 0.08) | 0.67 |  |
| Platelets (n=114) | 213 (153, 285) | 199(139,242) | 217(163,296) | 0.11 |  |
| **Medications** |  |  |  |  |  |
| Tocilizumab | 16 (14%) | 2 (8.7%) | 14 (15%) | 0.73 |  |
| Steroids | 37 (31%) | 4 (17%) | 33 (35%) | 0.14 |  |
| Hydroxychloroquine | 37 (31%) | 5 (22%) | 32 (34%) | 0.32 |  |
| **Other Autoantibodies** |  |  |  |  |  |
| Anti-CD209L IgM | 7 (6%) | 2 (9%) | 5 (5%) | 0.62 |  |
| Anti-IFNα IgG | 10 (9%) | 0 (0%) | 10 (11%) | 0.21 |  |
| Anti-ACE IgM | 21 (18%) | 1 (4%) | 21 (22%) | 0.07 |  |
| ^¶^ IQR= Interquartile range, BMI= body mass index, CAD= coronary artery disease, CHF= chronic heart failure, HTN= hypertension, ARB= Angiotensin II receptor blocker, ACEi= Angiotensin-converting enzyme inhibitor, WHO= World Health Organization, LOS= length of stay, WBC= white blood cell count, PMN= polymorphonuclear leukocytes, ^*^*P* < 0.05 | | | | | |

**Supplementary table 2.** Comparison of patients’ clinical characteristics based on the presence of anti-CD209L IgM autoantibody.

| **Patient Characteristics** | **Overall (n=118)**  **Median (IQR)** | **Detectable Anti-CD209L IgM (n=7)** | **Non-detectable IgM (n=111)** | ***P*-value** |  |
| --- | --- | --- | --- | --- | --- |
| **Age (years)** | 60 (50, 71) | 60 (56,72) | 60 (50, 71) | 0.63 |  |
| **Sex: Male, n (%)** | 66 (56%) | 7 (100%) | 59 (53%) | 0.02* |  |
| **Ethnicity, n (%)** |  |  |  | 0.91 |  |
| White | 31 (26%) | 2 (29%) | 29 (26%) |  |  |
| Black | 48 (41%) | 2 (29%) | 46 (41%) |  |  |
| Other | 35 (30%) | 3 (43%) | 32 (29%) |  |  |
| Hispanic | 27 (23%) | 3 (43%) | 24 (22%) | 0.21 |  |
| **BMI (n=106)** | 30.45 (26.23, 35.18) | 23.5(22.6,48.85) | 30.5(26.5,34.98) | 0.31 |  |
| **Max WHO Status** |  |  |  | 0.12 |  |
| Ventilation | 40 (34%) | 2 (29%) | 38 (34%) |  |  |
| Min O2 | 34 (29%) | 1 (14%) | 33 (30%) |  |  |
| HF Oxygen | 18 (15%) | 0 (0%) | 18 (16%) |  |  |
| Dead | 26 (22%) | 4 (57%) | 22 (20%) |  |  |
| **Max WHO severity, severe** | 66 (56%) | 6 (86%) | 60 (54%) | 0.13 |  |
| **Ventilation duration (h) (n=65)** | 449 (252, 919) | 348 (170,942) | 462(257,945) | 0.57 |  |
| **LOS (days), (n=115)** | 17 (8, 32) | 23(9,59) | 17(8,31) | 0.30 |  |
| **Comorbidities, n (%)** |  |  |  |  |  |
| Diabetes mellitus | 56 (47%) | 4 (57%) | 52 (47%) | 0.71 |  |
| **CAD** | 28 (24%) | 3 (43%) | 25(23%) | 0.35 |  |
| CHF | 27 (23%) | 2 (29%) | 25 (23%) | 0.66 |  |
| Lung disease | 31 (26%) | 2 (29%) | 29 (26%) | 1.00 |  |
| HTN | 76 (64%) | 5 (71%) | 71 (64%) | 1.00 |  |
| **ARB, n (%)** | 19 (16%) | 1 (14%) | 18 (16%) | 1.00 |  |
| **ACE inhibitors, n (%)** | 21 (17%) | 3 (43%) | 18 (16%) | 0.11 |  |
| **Either ARB or ACEi, n (%)** | 40 (33%) | 4 (57%) | 36 (32%) | 0.23 |  |
| **Other antihypertensive medications, n (%)** | 61 (52%) | 5 (71%) | 56 (51%) | 0.44 |  |
| **Admission Labs** |  |  |  |  |  |
| Creatinine | 1.1 (0.8, 1.73) | 1(0.8,2.7) | 1.1(0.8,1.7) | 0.97 |  |
| WBC (n=117) | 6.77 (5.27, 9.5) | 8.41(6.73,8.94) | 6.67(5.1,9.7) | 0.16 |  |
| Neutrophils (n=117) | 5.46 (3.6, 7.35) | 6.74(5.49,7.82) | 5.26(3.51,7.48) | 0.09 |  |
| Lymphocytes (n=117) | 0.83 (0.62, 1.34) | 0.67(0.47,0.94) | 0.89(0.62,1.36) | 0.24 |  |
| Monocytes (n=102) | 0.45 (0.31, 0.67) | 0.58(0.32,0.96) | 0.45(0.31,0.65) | 0.25 |  |
| Immature PMN (n=102) | 0.03 (0.02, 0.07) | 0.05(0.03,0.1) | 0.03(0.02,0.07) | 0.34 |  |
| Platelets (n=114) | 213 (153, 285) | 280(223,297) | 207(152,279) | 0.0508 |  |
| **Medications** |  |  |  |  |  |
| Tocilizumab | 16 (14%) | 1 (14%) | 15 (14%) | 1.00 |  |
| Steroids | 37 (31%) | 2 (29%) | 35 (32%) | 1.00 |  |
| Hydroxychloroquine | 37 (31%) | 3 (43%) | 34(31%) | 0.68 |  |
| **Other Autoantibodies** |  |  |  |  |  |
| Anti-CD209 IgM | 23 (20%) | 2 (28%) | 21 (19%) | 0.62 |  |
| Anti-IFNα IgG | 10 (9%) | 1 (14%) | 9 (8%) | 0.47 |  |
| Anti-ACE IgM | 21 (18%) | 1 (14%) | 20 (18%) | 1.00 |  |
| ^¶^ IQR= Interquartile range, BMI= body mass index, CAD= coronary artery disease, CHF= chronic heart failure, HTN= hypertension, ARB= Angiotensin II receptor blocker, ACEi= Angiotensin-converting enzyme inhibitor, WHO= World Health Organization, LOS= length of stay, WBC= white blood cell count, PMN= polymorphonuclear leukocytes, ^*^p< 0.05 | | | | | |

**Supplementary table 3**. Comparison of patients’ clinical characteristics based on the presence of anti-CD209 IgM and/or anti-CD209L IgM autoantibodies.

| **Patient Characteristics** | **Overall (n=118)**  **Median (IQR)** | **Detectable Anti-CD209L or CD209 IgM (n=28)**  **(2 patients double positive)** | **Non-detectable IgM (n=90)** | ***P*-value** |
| --- | --- | --- | --- | --- |
| **Age (years)** | 60 (50, 71) | 67 (56,73) | 59 (48, 69) | 0.03* |
| **Sex: Male, n (%)** | 66 (56%) | 18 (64%) | 48 (53%) | 0.39 |
| **Ethnicity, n (%)** |  |  |  | 0.17 |
| White | 31 (26%) | 12 (43%) | 19(21%) |  |
| Black | 48 (41%) | 8(29%) | 40(44%) |  |
| Other | 35 (30%) | 0(0%) | 3(3%) |  |
| Hispanic | 27 (23%) | 7 (25%) | 20 (22%) | 0.80 |
| **Max WHO Status** |  |  |  | 0.54 |
| Ventilation | 40 (34%) | 11 (39%) | 29(32%) |  |
| Min O2 | 34 (29%) | 5(18%) | 29(32%) |  |
| HF Oxygen | 18 (15%) | 5(18%) | 13(14%) |  |
| Dead | 26 (22%) | 7(25%) | 19(21%) |  |
| **Max WHO severity, severe** | 66 (56%) | 18(64%) | 48(53%) | 0.39 |
| **Ventilation duration (hours) (n=65)** | 449 (252, 919) | 398(188, 1010) | 466(274,890) | 0.78 |
| **BMI (n=106)** | 30.45 (26.23, 35.18) | 30.15(23.89,34.48) | 30.45(26.48,36.02) | 0.55 |
| **LOS (days), (n=115)** | 17 (8, 32) | 19(13,43) | 17(8,32) | 0.23 |
| **Comorbidities, n (%)** |  |  |  |  |
| Diabetes mellitus | 56 (47%) | 16(57%) | 40(44%) | 0.22 |
| CAD | 28 (24%) | 11(39%) | 17(19%) | 0.04* |
| CHF | 27 (23%) | 7(25%) | 20(22%) | 0.80 |
| Lung disease | 31 (26%) | 8(29%) | 23(26%) | 0.81 |
| HTN | 76 (64%) | 21(75%) | 55(61%) | 0.26 |
| **ARB, n (%)** | 19 (16%) | 6(21%) | 13(14%) | 0.39 |
| **ACE inhibitors, n (%)** | 21 (17%) | 8(29%) | 13(14%) | 0.10 |
| **Either ARB or ACEi, n (%)** | 40 (33%) | 14(50%) | 26(29%) | 0.07 |
| **Other antihypertensive medications, n (%)** | 61 (52%) | 17(61%) | 44(49%) | 0.29 |
| **Admission Labs** |  |  |  |  |
| Creatinine | 1.1 (0.8, 1.73) | 1.45(0.82,3) | 1.1(0.8,1.6) | 0.13 |
| WBC (n=117) | 6.77 (5.27, 9.5) | 7.12(6.01,8.91) | 6.69(5.08,10.15) | 0.56 |
| Neutrophils (n=117) | 5.46 (3.6, 7.35) | 5.81(4.35,7.18) | 5.16(3.48,7.65) | 0.38 |
| Lymphocytes (n=117) | 0.83 (0.62, 1.34) | 0.71(0.48,1.14) | 0.92(0.63,1.36) | 0.12 |
| Monocytes (n=102) | 0.45 (0.31, 0.67) | 0.42(0.30,0.58) | 0.47(0.31,0.68) | 0.57 |
| Immature PMN (n=102) | 0.03 (0.02, 0.07) | 0.03(0.02,0.05) | 0.03(0.02,0.08) | 0.84 |
| Platelets (n=114) | 213 (153, 285) | 206(148,275) | 213(159,298) | 0.44 |
| **Medications** |  |  |  |  |
| Tocilizumab | 16 (14%) | 3(11%) | 13(14%) | 0.76 |
| Steroids | 37 (31%) | 6(21%) | 31(34%) | 0.25 |
| Hydroxychloroquine | 37 (31%) | 7 (25%) | 30(33%) | 0.49 |
| **Other Autoantibodies** |  |  |  |  |
| Anti-IFNα IgG | 10 (9%) | 1 (4%) | 9 (10%) | 0.45 |
| Anti-ACE IgM | 21 (18%) | 2 (7%) | 19 (21%) | 0.15 |

| ^¶^ IQR= Interquartile range, BMI= body mass index, CAD= coronary artery disease, CHF= chronic heart failure, HTN= hypertension, ARB= Angiotensin II receptor blocker, ACEi= Angiotensin-converting enzyme inhibitor, WHO= World Health Organization, LOS= length of stay, WBC= white blood cell count, PMN= polymorphonuclear leukocytes, ^*^*P* < 0.05 |
| --- |

**Supplementary table 4.** Comparison of patients’ clinical characteristics based on the presence of anti-CD209 IgG autoantibody.

| **Patient Characteristics** | **Overall (n=118)**  **Median (IQR)** | **Detectable Anti-CD209 IgG (n=7)** | **Non-detectable IgG (n=111)** | ***P*-value** |
| --- | --- | --- | --- | --- |
| **Age (years)** | 60 (50, 71) | 57 (57,71) | 60 (50,71) | 0.49 |
| **Sex: Male, n (%)** | 66 (56%) | 2 (29%) | 64 (58%) | 0.13 |
| **Ethnicity, n (%)** |  |  |  | 0.22 |
| White | 31 (26%) | 2 (29%) | 29 (26%) |  |
| Black | 48 (41%) | 1 (14%) | 47 (42%) |  |
| Other | 35 (30%) | 4 (57%) | 35 (31%) |  |
| Hispanic | 27 (23%) | 3 (42%) | 24 (22%) | 0.21 |
| **BMI (n=106)** | 30.45 (26.23, 35.18) | 27.5 (22.63, 41.1) | 30.5 (26.38, 35.18) | 0.57 |
| **Max WHO Status** |  |  |  | 0.98 |
| Ventilation | 40 (34%) | 2 (29%) | 38 (34%) |  |
| Min O2 | 34 (29%) | 2 (29%) | 32 (29%) |  |
| HF Oxygen | 18 (15%) | 1 (14%) | 17 (15%) |  |
| Dead | 26 (22%) | 2 (29%) | 24 (22%) |  |
| **Max WHO severity, severe** | 66 (56%) | 4 (57%) | 62 (56%) | 0.95 |
| **Ventilation duration (hours) (n=65)** | 449 (252, 919) | 415 (280, 946) | 449 (244, 919) | 0.96 |
| **LOS (days), (n=115)** | 17 (8, 32) | 23 (7, 40) | 17 (8, 31) | 0.88 |
| **Comorbidities, n (%)** |  |  |  |  |
| Diabetes mellitus | 56 (47%) | 4 (57%) | 52 (47%) | 0.60 |
| CAD | 28 (24%) | 2 (29%) | 26 (23%) | 0.75 |
| CHF | 27 (23%) | 2 (29%) | 25 (23%) | 0.71 |
| Lung disease | 31 (26%) | 1 (14%) | 30 (27%) | 0.46 |
| HTN | 76 (64%) | 4 (57%) | 72 (65%) | 0.68 |
| **ARB, n (%)** | 19 (16%) | 0 (0%) | 19 (17%) | 0.23 |
| **ACE inhibitors, n (%)** | 21 (17%) | 1 (14%) | 20 (18%) | 0.80 |
| **Either ARB or ACEi, n (%)** | 40 (33%) | 1 (14%) | 39 (35%) | 0.26 |
| **Other antihypertensive medications, n (%)** | 61 (52%) | 2 (29%) | 59 (53%) | 0.21 |
| **Admission Labs** |  |  |  |  |
| Creatinine | 1.1 (0.8, 1.73) | 1.1 (0.8, 4.13) | 1.1 (0.8, 1.7) | 0.61 |
| WBC (n=117) | 6.77 (5.27, 9.5) | 7.77 (5.33, 8.94) | 6.75 (5.19, 9.7) | 0.77 |
| Neutrophils (n=117) | 5.46 (3.6, 7.35) | 6.54 (3.65, 7.82) | 5.41 (3.53, 7.48) | 0.66 |
| Lymphocytes (n=117) | 0.83 (0.62, 1.34) | 0.62 (0.4, 1.43) | 0.86 (0.63, 1.34) | 0.52 |
| Monocytes (n=102) | 0.45 (0.31, 0.67) | 0.54 (0.34, 0.61) | 0.45 (0.31, 0.67) | 0.61 |
| Immature PMN (n=102) | 0.03 (0.02, 0.07) | 0.04 (0.02,0.06) | 0.03 (0.02, 0.07) | 0.54 |
| Platelets (n=114) | 213 (153, 285) | 275 (126, 297) | 213 (153, 280) | 0.58 |
| **Medications** |  |  |  |  |
| Tocilizumab | 16 (14%) | 0 (0%) | 16 (14%) | 0.28 |
| Steroids | 37 (31%) | 2 (29%) | 35 (32%) | 0.87 |
| Hydroxychloroquine | 37 (31%) | 2 (29%) | 35 (32%) | 0.87 |
| **Other Autoantibodies** |  |  |  |  |
| Anti-CD209L IgM | 7 (6%) | 2 (9%) | 5 (5%) | 0.62 |
| Anti-CD209 IgM |  | 2 (29%) | 5 (71%) | 0.39 |
| Anti-IFNα IgG | 10 (9%) | 2 (9%) | 5 (5%) | 0.49* |
| Anti-ACE IgM | 21 (18%) | 0 (0%) | 7 (100%) | 0.20 |
| ^¶^ IQR= Interquartile range, BMI= body mass index, CAD= coronary artery disease, CHF= chronic heart failure, HTN= hypertension, ARB= Angiotensin II receptor blocker, ACEi= Angiotensin-converting enzyme inhibitor, WHO= World Health Organization, LOS= length of stay, WBC= white blood cell count, PMN= polymorphonuclear leukocytes, ANA= antinuclear antibodies, *^*^P* < 0.05 | | | | |

**Supplementary table 5.** Comparison of patients’ clinical characteristics based on the presence of anti-CD209L IgG autoantibody.

| **Patient Characteristics** | **Overall (n=118)**  **Median (IQR)** | **Detectable Anti-CD209L IgG (n=6)** | **Non-detectable IgG (n=112)** | ***P*-value** |  |
| --- | --- | --- | --- | --- | --- |
| **Age (years)** | 60 (50, 71) | 55 (45, 73) | 60 (50, 71) | 0.60 |  |
| **Sex: Male, n (%)** | 66 (56%) | 5 (83%) | 61 (55%) | 0.17 |  |
| **Ethnicity, n (%)** |  |  |  | 0.0003* |  |
| White | 31 (26%) | 3 (50%) | 28 (25%) |  |  |
| Black | 48 (41%) | 1 (17%) | 47 (42%) |  |  |
| Other | 35 (30%) | 2 (34%) | 37 (33%) |  |  |
| Hispanic | 27 (23%) | 1 (17%) | 26 (23%) | 0.86 |  |
| **BMI (n=106)** | 30.45 (26.23, 35.18) | 27.05 (22.76, 38.65) | 30.5 (26.43, 35.53) | 0.17 |  |
| **Max WHO Status** |  |  |  | 0.99 |  |
| Ventilation | 40 (34%) | 2 (34%) | 38 (34%) |  |  |
| Min O2 | 34 (29%) | 2 (34%) | 32 (29%) |  |  |
| HF Oxygen | 18 (15%) | 1 (17%) | 17 (16%) |  |  |
| Dead | 26 (22%) | 1 (17%) | 25 (22%) |  |  |
| **Max WHO severity, severe** | 66 (56%) | 3 (50%) | 63 (57%) | 0.76 |  |
| **Ventilation duration (hours) (n=65)** | 449 (252, 919) | 814 (394, 1384) | 432 (246, 906) | 0.27 |  |
| **LOS (days), (n=115)** | 17 (8, 32) | 18 (6, 52) | 17 98, 32) | 0.86 |  |
| **Comorbidities, n (%)** |  |  |  |  |  |
| Diabetes mellitus | 56 (47%) | 4 (67%) | 52 (47%) | 0.33 |  |
| CAD | 28 (24%) | 1 (17%) | 27 (24%) | 0.68 |  |
| CHF | 27 (23%) | 2 (34%) | 25 (22%) | 0.53 |  |
| Lung disease | 31 (26%) | 1 (17%) | 30 (27%) | 0.58 |  |
| HTN | 76 (64%) | 2 (34%) | 74 (66%) | 0.10 |  |
| **ARB, n (%)** | 19 (16%) | 0 (0%) | 19 (17%) | 0.27 |  |
| **ACE inhibitors, n (%)** | 21 (17%) | 2 (34%) | 19 (17%) | 0.31 |  |
| **Either ARB or ACEi, n (%)** | 40 (33%) | 2 (34%) | 38 (34%) | 0.98 |  |
| **Other antihypertensive medications, n (%)** | 61 (52%) | 2 (34%) | 59 (53%) | 0.36 |  |
| **Admission Labs** |  |  |  |  |  |
| Creatinine | 1.1 (0.8, 1.73) | 0.9 (0.75, 1.02) | 1.2 (0.8, 1.88) | 0.10 |  |
| WBC (n=117) | 6.77 (5.27, 9.5) | 9.1 (6.3, 12.94) | 6.73 (5.23, 9.29) | 0.18 |  |
| Neutrophils (n=117) | 5.46 (3.6, 7.35) | 7.71 (4.94, 10.08) | 5.28 (3.54, 7.41) | 0.15 |  |
| Lymphocytes (n=117) | 0.83 (0.62, 1.34) | 1.18 (0.55, 1.81) | 0.81 (0.61, 1.33) | 0.50 |  |
| Monocytes (n=102) | 0.45 (0.31, 0.67) | 0.59 (0.41, 0.95) | 0.45 (0.31, 0.64) | 0.17 |  |
| Immature PMN (n=102) | 0.03 (0.02, 0.07) | 0.06 (0.03, 0.18) | 0.03 (0.02, 0.07) | 0.24 |  |
| Platelets (n=114) | 213 (153, 285) | 268 (124, 462) | 213 (155, 280) | 0.43 |  |
| **Medications** |  |  |  |  |  |
| Tocilizumab | 16 (14%) | 1 (17%) | 15 (13%) | 0.82 |  |
| Steroids | 37 (31%) | 1 (17%) | 36 (32%) | 0.43 |  |
| Hydroxychloroquine | 37 (31%) | 1 (17%) | 36 (32%) | 0.43 |  |
| **Other Autoantibodies** |  |  |  |  |  |
| Anti-CD209 IgM | 23 (20%) | 1 (17%) | 22 (20%) | 0.86 |  |
| Anti-CD209L IgM |  | 1 (17%) | 6 (5%) | 0.25 |  |
| Anti-IFNα IgG | 10 (9%) | 0 (0%) | 10 (9%) | 0.44 |  |
| Anti-ACE IgM | 21 (18%) | 1 (17%) | 20 (18%) | 0.94 |  |
| ^¶^ IQR= Interquartile range, BMI= body mass index, CAD= coronary artery disease, CHF= chronic heart failure, HTN= hypertension, ARB= Angiotensin II receptor blocker, ACEi= Angiotensin-converting enzyme inhibitor, WHO= World Health Organization, LOS= length of stay, WBC= white blood cell count, PMN= polymorphonuclear leukocytes, ^*^*P* < 0.05 | | | | | |

**Supplementary table 6.** Comparison of patients’ clinical characteristics based on the presence of either anti-CD209 IgM, anti-CD209L IgM, anti-CD209 IgG and/or anti-CD209L IgG autoantibody.

| **Patient Characteristics** | **Overall (n=118)**  **Median (IQR)** | **Detectable anti-CD209/CD209L IgM/IgG (n=38)** | **Non-detectable anti-CD209/CD209L IgM/IgG (n=80)** | ***P*-value** |  |
| --- | --- | --- | --- | --- | --- |
| **Age (years)** | 60 (50,71) | 68 (56, 73) | 59 (47, 68) | 0.02* |  |
| **Sex: Male, n (%)** | 66 (56%) | 23 (61%) | 43 (54%) | 0.49 |  |
| **Ethnicity, n (%)** |  |  |  | 0.02* |  |
| White | 31 (26%) | 15 (40%) | 19 (20%) |  |  |
| Black | 48 (41%) | 9 (24%) | 39 (49%) |  |  |
| Other | 35 (30%) | 14 (37%) | 25 (32%) |  |  |
| Hispanic | 27 (23%) |  |  |  |  |
| **BMI (n=106)** | 30.45 (26.23, 35.18) | 28.9 (24.02, 34.23) | 20.55 (26.76, 36.28) | 0.18 |  |
| **Max WHO Status** |  |  |  | 0.88 |  |
| Ventilation | 40 (34%) | 12 (32%) | 28 (35%) |  |  |
| Min O2 | 34 (29%) | 10 (26%) | 24 (30%) |  |  |
| HF Oxygen | 18 (15%) | 6 (16%) | 12 (15%) |  |  |
| Dead | 26 (22%) | 10 (26%) | 16 (20%) |  |  |
| **Max WHO severity, severe** | 66 (56%) | 22 (58%) | 44 (55%) | 0.77 |  |
| **Ventilation duration (hours) (n=65)** | 449 (252, 919) | 398 (227, 1010) | 467 (255, 890) | 0.88 |  |
| **LOS (days), (n=115)** | 17 (8, 32) | 18 (10-35) | **17 (8, 32)** | 0.54 |  |
| **Comorbidities, n (%)** |  |  |  |  |  |
| Diabetes mellitus | 56 (47%) | 20 (53%) | 36 (45%) | 0.44 |  |
| CAD | 28 (24%) | 12 (32%) | 16 (20%) | 0.17 |  |
| CHF | 27 (23%) | 9 (24%) | 18 (23%) | 0.89 |  |
| Lung disease | 31 (26%) | 10 (36%) | 21 (27%) | 0.99 |  |
| HTN | 76 (64%) | 25 (66%) | 51 (64%) | 0.83 |  |
| **ARB, n (%)** | 19 (16%) | 6 (16%) | 13 (26%) | 0.95 |  |
| **ACE inhibitors, n (%)** | 21 (17%) | 8 (21%) | 13 (16%) | 0.52 |  |
| **Either ARB or ACEi, n (%)** | 40 (33%) | 14 (37%) | 26 (33%) | 0.65 |  |
| **Other antihypertensive medications, n (%)** | 61 (52%) | 19 (50%) | 42 (53%) | 0.80 |  |
| **Admission Labs** |  |  |  |  |  |
| Creatinine | 1.1 (0.8, 1.73) | 1.15 (0.88, 2.7) | 1.1 (0.8, 1.6) | 0.21 |  |
| WBC (n=117) | 6.77 (5.27, 9.5) | 6.78 (5.27, 9.0) | 6.77 (5.23, 10.14) | 0.68 |  |
| Neutrophils (n=117) | 5.46 (3.6, 7.35) | 5.63 (3.85, 7.42) | 5.24 (3.49, 7.65) | 0.52 |  |
| Lymphocytes (n=117) | 0.83 (0.62, 1.34) | 0.73 (0.52, 1.28) | 0.92 (0.63, 1.35) | 0.27 |  |
| Monocytes (n=102) | 0.45 (0.31, 0.67) | 0.48 (0.33, 0.661) | 0.45 (0.31, 0.67) | 0.54 |  |
| Immature PMN (n=102) | 0.03 (0.02, 0.07) | 0.03 (0.02, 0.06) | 0.03 (0.02, 0.07) | 0.99 |  |
| Platelets (n=114) | 213 (153, 285) | 216 (145, 287) | 213 (161, 285) | 0.77 |  |
| **Medications** |  |  |  |  |  |
| Tocilizumab | 16 (14%) | 4 (11%) | 12 (15%) | 0.50 |  |
| Steroids | 37 (31%) | 9 (24%) | 28 (35%) | 0.22 |  |
| Hydroxychloroquine | 37 (31%) | 8 (21%) | 29 (36%) | 0.10 |  |
| **Other Autoantibodies** |  |  |  |  |  |
| Anti-IFNα IgG | 10 (9%) | 3 (8%) | 7 (9%) | 0.88 |  |
| Anti-ACE IgM | 21 (18%) | 3 (7%) | 18 (23%) | 0.05 |  |
| ^¶^ IQR= Interquartile range, BMI= body mass index, CAD= coronary artery disease, CHF= chronic heart failure, HTN= hypertension, ARB= Angiotensin II receptor blocker, ACEi= Angiotensin-converting enzyme inhibitor, WHO= World Health Organization, LOS= length of stay, WBC= white blood cell count, PMN= polymorphonuclear leukocytes, ^*^p< 0.05 | | | | | |

**Supplementary Table 7.** Comparison of patients’ clinical characteristics based on the presence of anti-IFNα IgG autoantibody.

| **Patient Characteristics** | **Overall (n=118)**  **Median (IQR)** | **Detectable Anti-IFNα IgG (n=10)** | **Non-detectable IgG (n=108)** | **p-value** |  |
| --- | --- | --- | --- | --- | --- |
| **Age (years)** | 60 (50,71) | 66(56, 73) | 60(50,71) | 0.27 |  |
| **Sex: Male, n (%)** | 66 (56%) | 6 (60%) | 60 (56%) | 1.00 |  |
| **Ethnicity, n (%)** |  |  |  | 0.41 |  |
| White | 31 (26%) | 1 (10%) | 30 (28%) |  |  |
| Black | 48 (41%) | 4 (40%) | 44(41%) |  |  |
| Other | 35 (30%) | 4 (40%) | 31 (29%) |  |  |
| Hispanic | 27 (23%) | 3 (30%) | 24 (22%) | 0.60 |  |
| **BMI (n=106)** | 30.45 (26.23, 35.18) | 29(24.9,38) | 30.5(26.3,35) | 0..96 |  |
| **Max WHO Status** |  |  |  | 0.02* |  |
| Ventilation | 40 (34%) | 5 (50%) | 35 (32%) |  |  |
| Min O2 | 34 (29%) | 0 (0%) | 34 (32%) |  |  |
| HF Oxygen | 18 (15%) | 0 (0%) | 018 (17%) |  |  |
| Dead | 26 (22%) | 5 (50%) | 21 (19%) |  |  |
| **Max WHO severity, severe** | 66 (56%) | 10 (100%) | 56 (52%) | 0.0023* |  |
| **Ventilation duration (hours) (n=65)** | 449 (252, 919) | 559(346,1004) | 414(216,879) | 0.23 |  |
| **LOS (days), (n=115)** | 17 (8, 32) | 29(25,50) | 15(8,31) | 0.005 |  |
| **Comorbidities, n (%)** |  |  |  |  |  |
| Diabetes mellitus | 56 (47%) | 6 (60%) | 50 (46%) | 0.51 |  |
| CAD | 28 (24%) | 0 (0%) | 28 (26%) | 0.11 |  |
| CHF | 27 (23%) | 0 (0%) | 27 (25%) | 0.11 |  |
| Lung disease | 31 (26%) | 3(30%) | 28(26%) | 0.72 |  |
| HTN | 76 (64%) | 8 (80%) | 68(63%) | 0.49 |  |
| **ARB, n (%)** | 19 (16%) | 2 (20%) | 17 (16%) | 0.66 |  |
| **ACE inhibitors, n (%)** | 21 (17%) | 1 (10%) | 20 (29%) | 0.69 |  |
| **Either ARB or ACEi, n (%)** | 40 (33%) | 3 (30%) | 37 (34%) | 1.00 |  |
| **Other antihypertensive medications, n (%)** | 61 (52%) | 5 (50%) | 56(52%) | 1.00 |  |
| **Admission Labs** |  |  |  |  |  |
| Creatinine | 1.1 (0.8, 1.73) | 1.3(0.96,1.98) | 1.1(0.8,1.7) | 0.54 |  |
| WBC (n=117) | 6.77 (5.27, 9.5) | 9.9(8.19,16.64) | 6.55(5.07,9.04) | 0.0013 |  |
| Neutrophils (n=117) | 5.46 (3.6, 7.35) | 8.82(6.85,14.34) | 5.16(3.49,6.8) | 0.0005* |  |
| Lymphocytes (n=117) | 0.83 (0.62, 1.34) | 0.64(0.47,0.87) | 0.9(0.63,1.39) | 0.05 |  |
| Monocytes (n=102) | 0.45 (0.31, 0.67) | 0.48(0.32,1.22) | 0.45(0.31,0.62) | 0.46 |  |
| Immature PMN (n=102) | 0.03 (0.02, 0.07) | 0.12(0.08,0.27) | 0.03(0.02,0.05) | 0.0002* |  |
| Platelets (n=114) | 213 (153, 285) | 274(225,321) | 207(151,279) | 0.02* |  |
| **Medications** |  |  |  |  |  |
| Tocilizumab | 16 (14%) | 1(10%) | 15(14%) | 1.00 |  |
| Steroids | 37 (31%) | 7(70%) | 30(28%) | 0.01* |  |
| Hydroxychloroquine | 37 (31%) | 2(20%) | 35(32%) | 0.50 |  |
| **Other Autoantibodies** |  |  |  |  |  |
| Anti-CD209 IgM | 23 (20%) | 0 (0%) | 23 (21%) | 0.21 |  |
| Anti-CD209L IgM | 7 (6%) | 1 (14%) | 6 (6%) | 0.47 |  |
| Anti-CD209 or CD209L | 28 (24%) | 1 (4%) | 27 (25%) | 0.45 |  |
| Anti-ACE2 IgM | 21 (18%) | 3 (14%) | 18 (17%) | 0.38 |  |
| ^¶^ IQR= Interquartile range, BMI= body mass index, CAD= coronary artery disease, CHF= chronic heart failure, HTN= hypertension, ARB= Angiotensin II receptor blocker, ACEi= Angiotensin-converting enzyme inhibitor, WHO= World Health Organization, LOS= length of stay, WBC= white blood cell count, PMN= polymorphonuclear leukocytes, ^*^*P* < 0.05 | | | | | |

**Supplementary table 8.** Comparison of patients’ clinical characteristics based on anti-ACE2 IgM autoantibody status.

| **Patient Characteristics** | **Overall (n=118)**  **Median (IQR)** | **Anti-ACE2 IgM positive(n=21)** | **Anti-ACE2 IgM negative (n=97)** |  | |
| --- | --- | --- | --- | --- | --- |
| **Age (years)** | 60 (50,71) | 59(53,69) | 60(50,71) | 0.89 | |
| **Sex: Male, n (%)** | 66 (56%) | 14 (67%) | 52 (54%) | 0.34 | |
| **Ethnicity, n (%)** |  |  |  | 0.18 | |
| White | 31 (26%) | 4 (19%) | 27 (28%) |  | |
| Black | 48 (41%) | 10 (48%) | 38 (39%) |  | |
| Other | 35 (30%) | 5 (24%) | 30 (31%) |  | |
| Hispanic | 27 (23%) | 3 (14%) | 24 (25%) | 0.28 | |
| **BMI (n=106)** | 30.45 (26.23, 35.18) | 30.9 (25.63,42.88) | 30.4(26.38,34.76) | 0.41 | |
| **Max WHO Status** |  |  |  | 0.0016* | |
| Ventilation | 40 (34%) | 14 (67%) | 26 (27%) |  | |
| Min O2 | 34 (29%) | 2 (10%) | 32 (33%) |  | |
| HF Oxygen | 18 (15%) | 0 (0%) | 18 (19%) |  | |
| Dead | 26 (22%) | 5 (24%) | 21 (22%) |  | |
| **Max WHO severity, severe** | 66 (56%) | 19 (91%) | 47 (49%) | 0.0005* | |
| **Ventilation duration (hours) (n=65)** | 449 (252, 919) | 665 (449,979) | 356(211,883) | 0.047* | |
| LOS (days), (n=115) | 17 (8, 32) | 37(27,60) | 14(8,25) | <0.0001* | |
| **Comorbidities, n (%)** |  |  |  |  | |
| Diabetes mellitus | 56 (47%) | 12 (57%) | 44 (45%) | 0.35 | |
| CAD | 28 (24%) | 4 (19%) | 24 (25%) | 0.78 | |
| CHF | 27 (23%) | 8 (38%) | 19 (20%) | 0.09 | |
| Lung disease | 31 (26%) | 3 (14%) | 28 (29%) | 0.27 | |
| HTN | 76 (64%) | 17 (81%) | 59 (61%) | 0.13 | |
| **ARB, n (%)** | 19 (16%) | 3 (14%) | 16 (17%) | 1.00 | |
| **ACE inhibitors, n (%)** | 21 (17%) | 2 (10%) | 19 (20%) | 0.36 | |
| **Either ARB or ACEi, n (%)** | 40 (33%) | 5 (24%) | 35 (36%) | 0.32 | |
| **Other antihypertensive medications, n (%)** | 61 (52%) | 13 (62%) | 48 (50%) | 0.34 | |
| **Admission Labs** |  |  |  |  | |
| Creatinine | 1.1 (0.8, 1.73) | 1.2(1,1.55) | 1.1(0.8,1.9) | 0.20 | |
| WBC (n=117) | 6.77 (5.27, 9.5) | 6.69(5.7,7.91) | 6.83(5.13,10.02) | 0.77 | |
| Neutrophils (n=117) | 5.46 (3.6, 7.35) | 5.24(4.25,6.39) | 5.51(3.50,7.65) | 0.98 | |
| Lymphocytes (n=117) | 0.83 (0.62, 1.34) | 0.81(0.58,1.1) | 0.86(0.62,1.4) | 0.31 | |
| Monocytes (n=102) | 0.45 (0.31, 0.67) | 0.42(0.32,0.59) | 0.45(0.31,0.67) | 0.68 | |
| Immature PMN (n=102) | 0.03 (0.02, 0.07) | 0.02 (0.02,0.03) | 0.04(0.02,0.07) | 0.12 | |
| Platelets (n=114) | 213 (153, 285) | 182(146,221) | 221(158,297) | 0.09 | |
| **Medications** |  |  |  |  | |
| Tocilizumab | 16 (14%) | 6 (29%) | 10 (10%) | 0.04* | |
| Steroids | 37 (31%) | 9 (43%) | 28 (29%) | 0.30 | |
| Hydroxychloroquine | 37 (31%) | 15 (71%) | 22 (23%) | <0.0001* | |
| **Other Autoantibodies** |  |  |  |  | |
| Anti-CD209 IgM | 23 (20%) | 1 (5%) | 22 (23%) | 0.07 | |
| Anti-CD209L IgM | 7 (6%) | 1 (5%) | 6 (6%) | 1.00 | |
| Anti-CD209 or CD209L IgM | 28 (24%) | 2 (10%) | 26 (27%) | 0.15 | |
| Anti-IFNα IgG | 10 (9%) | 3 (14%) | 7 (7%) | 0.38 | |
| ^¶^ IQR= Interquartile range, BMI= body mass index, CAD= coronary artery disease, CHF= chronic heart failure, HTN= hypertension, ARB= Angiotensin II receptor blocker, ACEi= Angiotensin-converting enzyme inhibitor, WHO= World Health Organization, LOS= length of stay, WBC= white blood cell count, PMN= polymorphonuclear leukocytes, ^*^p< 0.05 | | | | |  |

**Supplementary table 9.** Comparison of patients’ clinical characteristics based on the presence of anti-ACE2 IgG autoantibody.

| **Patient Characteristics** | **Overall (n=103)**  **Median (IQR)** | **Detectable Anti-ACE2 IgG (n=15)** | **Non-detectable IgG (n=88)** | ***P*-value** |  |
| --- | --- | --- | --- | --- | --- |
| **Age (years)** | 60 (51, 70) | 59 (56, 68) | 60 (50, 71) | 0.93 |  |
| **Sex: Male, n (%)** | 57 (55%) | 10 (67%) | 47 (53%) | 0.34 |  |
| **Ethnicity, n (%)** |  |  |  | 0.10 |  |
| White | 26 (25%) | 3 (20%) | 23 (26%) |  |  |
| Black | 41 (40%) | 7 (47%) | 34 (39%) |  |  |
| Other | 35 (30%) | 5 (34%) | 31 (35%) |  |  |
| Hispanic | 24 (23%) | 1 (7%) | 23 (26%) | 0.14 |  |
| **BMI (n=93)** | 30.45 (26.23, 35.18) | 27.9 (23.9, 24.9) | 30.5 (27.03, 35.75) | 0.14 |  |
| **Max WHO Status** |  |  |  | 0.56 |  |
| Ventilation | 40 (39%) | 7 (47%) | 33 (38%) |  |  |
| Min O2 | 20 (20%) | 4 (27%) | 16 (18%) |  |  |
| HF Oxygen | 18 (18%) | 1 (7%) | 17 (19%) |  |  |
| Dead | 25 (24%) | 3 (20%) | 22 (25%) |  |  |
| **Max WHO severity, severe** | 65 (63%) | 10 (67%) | 55 (63%) | 0.76 |  |
| **Ventilation duration (hours) (n=64)** | 431 (250, 932) | 695 *370, 847) | 399 (240, 948) | 0.31 |  |
| **LOS (days), (n=100)** | 20 (8, 36) | 28 (9, 48) | 18 (8, 34) | 0.13 |  |
| **Comorbidities, n (%)** |  |  |  |  |  |
| Diabetes mellitus | 53 (52%) | 7 (47%) | 46 (52%) | 0.67 |  |
| CAD | 23 (22%) | 3 (20%) | 20 (23%) | 0.81 |  |
| CHF | 24 (23%) | 7 (47%) | 17 (19%) | 0.02* |  |
| Lung disease | 27 (26%) | 2 (13%) | 25 (28%) | 0.22 |  |
| HTN | 66 (64%) | 9 (60%) | 57 (65%) | 0.72 |  |
| **ARB, n (%)** | 17 (17%) | 1 (7%) | 16 (18%) | 0.27 |  |
| **ACE inhibitors, n (%)** | 17 (17%) | 2 (13%) | 15 (17%) | 0.72 |  |
| **Either ARB or ACEi, n (%)** | 34 (33%) | 3 (20%) | 31 (36%) | 0.25 |  |
| **Other antihypertensive medications, n (%)** | 54 (52%) | 8 (53%) | 46 (52%) | 0.94 |  |
| **Admission Labs** |  |  |  |  |  |
| Creatinine | 1.16 (0.8, 1.7) | 1.2 (0.9, 1.3) | 1.13 (0.8, 1.9) | 0.71 |  |
| WBC (n=102) | 6.8 (5.34, 9.23) | 7.54 (6.11, 9.21) | 6.73 (5.3, 9.29) | 0.69 |  |
| Neutrophils (n=102) | 5.48 (3.78, 7.69) | 5.78 (4.69, 8.82) | 5.36 (3.65, 7.75) | 0.60 |  |
| Lymphocytes (n=102) | 0.81 (0.62, 1.25) | 0.89 (0.63, 1.23) | 0.81 (0.6, 1.31) | 0.99 |  |
| Monocytes (n=89) | 0.44 (0.31, 0.61) | 0.49 (0.32, 0.55) | 0.43 (0.30, 0.62) | 0.44 |  |
| Immature PMN (n=89) | 0.03 (0.02, 0.07) | 0.03 (0.02, 0.05) | 0.03 (0.02, 0.08) | 0.86 |  |
| Platelets (n=99) | 213 (152, 279) | 212 (148, 261) | 210 (152, 291) | 0.51 |  |
| **Medications** |  |  |  |  |  |
| Tocilizumab | 16 (16%) | 3 (20%) | 13 (15%) | 0.61 |  |
| Steroids | 33 (32%) | 4 (27%) | 29 (33%) | 0.63 |  |
| Hydroxychloroquine | 37 (36%) | 7 (47%) | 30 (34%) | 0.35 |  |
| **Other Autoantibodies** |  |  |  |  |  |
| Anti-CD209 IgM | 21 (20%) | 2 (13%) | 19 (22%) | 0.46 |  |
| Anti-CD209L IgM | 7 (7%) | 1 (7%) | 6 (7%) | 0.98 |  |
| Anti-CD209 or CD209L | 26 (25%) | 3 (20%) | 23 (26%) | 0.61 |  |
| Anti-IFNα IgG | 9 (9%) | 0 (0%) | 9 (10%) | 0.19 |  |
| Anti-ACE2 IgM | 20 (19%) | 6 (40%) | 14 (16%) | 0.03* |  |
| ^¶^ IQR= Interquartile range, BMI= body mass index, CAD= coronary artery disease, CHF= chronic heart failure, HTN= hypertension, ARB= Angiotensin II receptor blocker, ACEi= Angiotensin-converting enzyme inhibitor, WHO= World Health Organization, LOS= length of stay, WBC= white blood cell count, PMN= polymorphonuclear leukocytes, ^*^*P* < 0.05 | | | | | |

**Supplementary table 10.** Comparison of hospitalized patients’ baseline characteristics per COVID-19 infection severity.

| **Patient Characteristics** | **Overall (n=118)**  **Median (IQR)** | **Severe Disease (n=66)** | **Moderate Disease (n=52)** | ***P*-value** |  |
| --- | --- | --- | --- | --- | --- |
| **Age (years)** | 60 (50, 71) | 63 (56,80) | 55 (44,79) | 0.014 |  |
| **Sex: Male, n (%)** | 66 (56%) | 38 (58%) | 28 (54%) | 0.71 |  |
| **Ethnicity, n (%)** |  |  |  | 0.42 |  |
| White | 31 (26%) | 20 (30%) | 11 (21%) |  |  |
| Black | 48 (41%) | 28 (42%) | 20 (39%) |  |  |
| Other | 35 (30%) | 16 (24%) | 19 (37%) |  |  |
| Hispanic | 27 (23%) | 13 (20%) | 14 (27%) | 0.35 |  |
| **BMI (n=106)** | 30.45 (26.23, 35.18) | 30.4 (26.2,37.35) | 30.5(26.15,37.1) | 0.34 |  |
| **Ventilation duration (hours) (n=65)** | 449 (252, 919) | 449 (252, 919) | N/A | N/A |  |
| **LOS (days), (n=115)** | 17 (8, 32) | 28(20,47) | 9(4,14) | <0.001* |  |
| **Comorbidities, n (%)** |  |  |  |  |  |
| Diabetes mellitus | 56 (47%) | 39(59%) | 17(33%) | 0.0054* |  |
| CAD | 28 (24%) | 19(29%) | 9(17%) | 0.19 |  |
| CHF | 27 (23%) | 21 (32%) | 6 (12%) | 0.01* |  |
| Lung disease | 31 (26%) | 20(30%) | 11(21%) | 0.30 |  |
| HTN | 76 (64%) | 49 (74%) | 27 (52%) | 0.02* |  |
| **ARB, n (%)** | 19 (16%) | 13(20%) | 6(12%) | 0.32 |  |
| **ACE inhibitors, n (%)** | 21 (17%) | 14(21%) | 7(14%) | 0.34 |  |
| **Either ARB or ACEi, n (%)** | 40 (33%) | 27(41%) | 13(25%) | 0.08 |  |
| **Other antihypertensive medications, n (%)** | 61 (52%) | 40(61%) | 21(40%) | 0.04* |  |
| **Admission Labs** |  |  |  |  |  |
| Creatinine | 1.1 (0.8, 1.73) | 1.4(1.0,2.2) | 0.85(0.7,1.3) | <0.0001* |  |
| WBC (n=117) | 6.77 (5.27, 9.5) | 6.75(5.57,9.38) | 6.84(4.96,9.64) | 0.62 |  |
| Neutrophils (n=117) | 5.46 (3.6, 7.35) | 5.48(4.02,8.57) | 5.16(3.22,6.66) | 0.08 |  |
| Lymphocytes (n=117) | 0.83 (0.62, 1.34) | 0.72(0.5,1.06) | 1.24(0.69,1.51) | <0.0001* |  |
| Monocytes (n=102) | 0.45 (0.31, 0.67) | 0.38(0.27,0.59) | 0.49(0.36,0.74) | 0.03* |  |
| Immature PMN (n=102) | 0.03 (0.02, 0.07) | 0.03(0.02,0.1) | 0.03(0.02,0.06) | 0.43 |  |
| Platelets (n=114) | 213 (153, 285) | 204(153,263) | 231(176,314) | 0.19 |  |
| **Medications** |  |  |  |  |  |
| Tocilizumab | 16 (14%) | 15(23%) | 1(2%) | 0.0008* |  |
| Steroids | 37 (31%) | 31(47%) | 6(12%) | <0.0001* |  |
| Hydroxychloroquine | 37 (31%) | 31(47%) | 6(12%) | <0.0001* |  |
| **Other Autoantibodies** |  |  |  |  |  |
| Anti-CD209 IgM | 23 (20%) | 14 (21%) | 9 (17%) | 0.65 |  |
| Anti-CD209L IgM | 7 (6%) | 6 (9%) | 1 (2%) | 0.13 |  |
| Anti-CD209 or CD209L IgM | 28 (24%) | 18 (27%) | 10 (19%) | 0.39 |  |
| Anti-IFNα IgG | 10 (9%) | 10 (15%) | 0 (0%) | 0.0023* |  |
| Anti-ACE IgM | 21 (18%) | 19 (29%) | 2 (4%) | 0.0005* |  |
| Either Ab | 53 (45%) | 41 (62%) | 12 (23%) | <0.0001* |  |
| ^¶^ IQR= Interquartile range, N/A= non-applicable, BMI= body mass index, CAD= coronary artery disease, CHF= chronic heart failure, HTN= hypertension, ARB= Angiotensin II receptor blocker, ACEi= Angiotensin-converting enzyme inhibitor, WHO= World Health Organization, LOS= length of stay, WBC= white blood cell count, PMN= polymorphonuclear leukocytes, ^*^*P* < 0.05 | | | | | |
